# Copper-catalyzed aerobic oxidative radical alkoxycyclization of tryptamines to access 3-alkoxypyrroloindolines†

**DOI:** 10.1039/d1ra02679h

**Published:** 2021-05-19

**Authors:** Wei Wang, Jun-Rong Song, Zhi-Yao Li, Ting Zhong, Qin Chi, Hai Ren, Wei-Dong Pan

**Affiliations:** School of Pharmaceutical Sciences, Guizhou University Huaxi Avenue South Guiyang 550025 P. R. China wdpan@163.com; State Key Laboratory of Functions and Applications of Medicinal Plants, Guizhou Medical University, The Key Laboratory of Chemistry for Natural Products of Guizhou Province and Chinese Academy of Sciences Guiyang 550014 China renh@gzcnp.cn

## Abstract

We report a copper-catalyzed alkoxycyclization of tryptamine derivatives with O_2_ as the sole oxidant, leading to a variety of C3a-alkoxypyrroloindolines in good yields with high diastereoselectivities. This reaction involves an interesting double catalytic cycle in which copper-catalyzed carboamination cyclization is favored to form the C-3 radical pyrrolidinoindoline intermediate, then a copper-catalytic radical alkoxylation reaction proceeds smoothly.

## Abstract 2

An oxazoline/copper-catalyzed cascade carboamination alkoxylation of substituted tryptamine under mild eco-friendly O_2_ oxidation conditions was reported.

## Graphical abstract

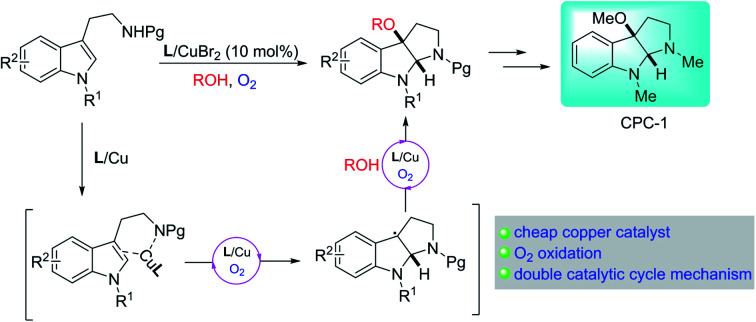

Pyrrolidino[2,3-*b*]indoline is an important heterocyclic core skeleton that exists in numerous biologically active natural products and pharmaceutical molecules.^1^ Cyclotryptamine type molecules which are oxygenated at the C3a position are especially outstanding due to their prominent bioactivity profiles,^2^ various applications in biological probes^3^ and chiral catalysts.^4^

As direct access to these complex products, the development of C3a-oxygenation/cyclization reactions of tryptamine or tryptophan derivatives has attracted extensive interest from synthetic chemists. Recently, some remarkable efforts have contributed to the one-step assembly of 3-hydroxyl,^5^ acetoxyl,^6^ peroxyl^7^ and other oxygenated^8^ pyrroloindolines through oxidative cyclization of tryptophan substrates. However, by utilizing a similar strategy, the direct synthesis of 3-alkoxyl pyrroloindolines remains less developed. In 2020, Zhong *et al.*^9^ reported the first example of alkoxycyclization of tryptamine derivatives using molecular iodine catalyst with *tert*-butyl hydroperoxide as the oxidant. None of the other studies, like using transition-metal catalysts, have been described yet.

Copper salts, which are inexpensive and easily accessible, have been widely used in organic synthesis as catalysts. Copper(ii)-promoted radical intramolecular carboamination of alkene has proven to be an effective means toward the synthesis of N-fused heterocycles.^10^ Recent reports have utilized this strategy toward the cyclization and radical alkylation, aromatization and aminooxygenation of alkene.^10^ However, due to the difficulty in homolytic breakage of the oxygen–hydrogen bond in alcohols with a high bond dissociation energy (BDE is *ca.*105 kcal mol^−1^),^11^ the related direct cyclization and radical alkoxylation of carbon–carbon double bond with copper catalysts is still unknown. Inspired by the relevant research of copper-catalyzed radical alkoxylation reaction,^12^ we assume that if the catalytic carboamination and radical alkoxylation tandem reaction could be realized by a single copper catalyst, which will represent as a new effective protocol for the direct construction of alkoxyl-containing N-fused heterocycles. Herein, we report an oxazoline/copper-catalyzed cascade carboamination alkoxylation of substituted tryptamine under mild eco-friendly O_2_ oxidation conditions, which facilitate the construction of the 3-alkoxyl pyrroloindolinese motif in good yield with good to excellent levels of diastereoselectivity (Scheme 1).

**Scheme 1:**
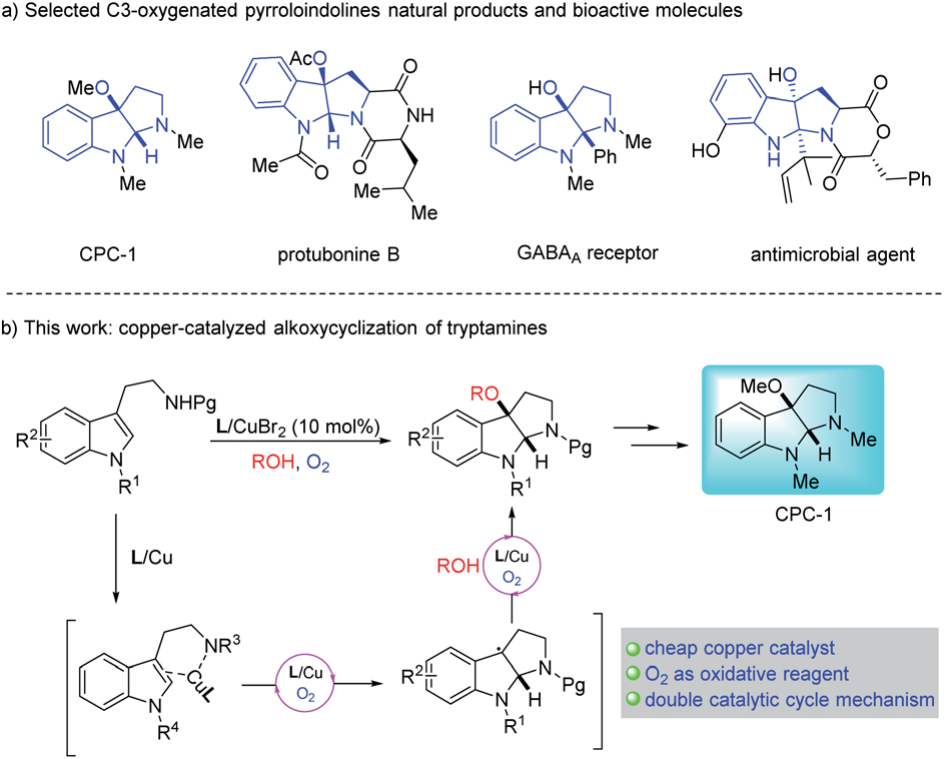
Copper-catalyzed cyclization and alkoxylation of tryptamines.

In our studies, the commercial easily available *N*-methyl tryptamine 1a was chosen as model substrate. Initially, 10 mol% of metal salt CuBr_2_ was used as catalyst, the 3-alkoxylation product 2a was obtained as 38% yield with 14/1 dr (Table 1, entry 1). When the diimine ligand L1 was added, only 28% yield of desired product was obtained (Table 1, entry 2). Interestingly, when the bisbenzoxazoline L2/CuBr_2_ was used, the yield of the reaction was obtained in 45% with 20/1 dr (Table 1, entry 3). The bisbenzothiazoline L3 and dibenzyl-modified bisbenzoxazoline L4 failed to improve the reaction (Table 1, entries 4–5). Attempts to improve the yield by further screening of copper salts were not successful (Table 1, entries 6–10). A better result was obtained by increasing the solvent of methanol resulting in 71% yield with 20/1 dr (Table 1, entry 11). When the reaction was conducted at the air atmosphere, the yield decreased greatly (Table 1, entry 12). Therefore, the employment of L2/CuBr_2_ (12/10 mol%) in 4 mL MeOH at 50 °C was selected as the optimal conditions for this reaction.

**Table 1:** Conditions optimization for alkoxylation^a^

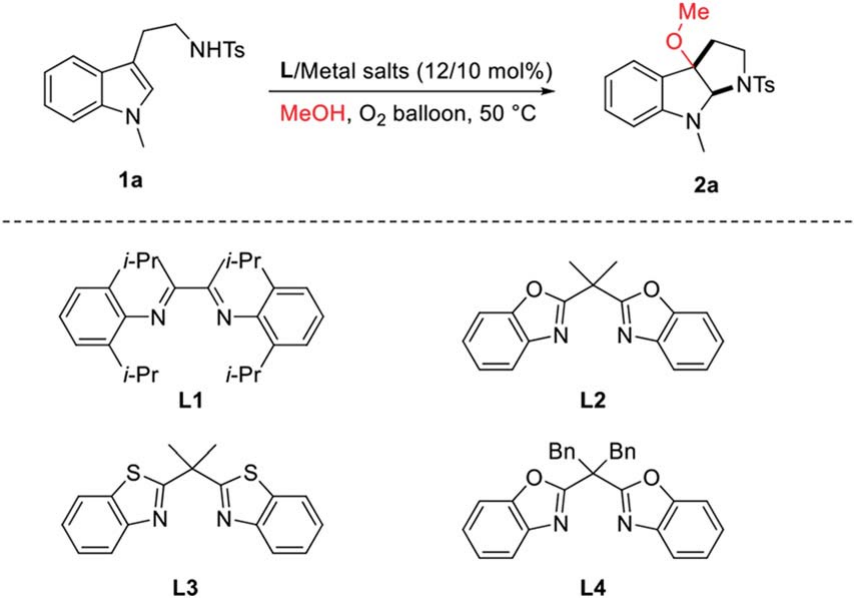				
Entry	Metal salts	Ligand	Yield^b^ (%)	Dr^c^
1	CuBr_2_	—	38	14/1
2	CuBr_2_	L1	28	>20/1
3	CuBr_2_	L2	45	>20/1
4	CuBr_2_	L3	24	13/1
5	CuBr_2_	L4	35	8/1
6	Cu(OTf)_2_	L2	Trace	—
7	CuO	L2	nr	—
8	Cu(OAc)_2_	L2	nr	—
9	Cu(ClO_4_)_2_	L2	nr	—
10	CuCl_2_	L2	15	8/1
11^d^	CuBr_2_	L2	71	>20/1
12^d^^,^^e^	CuBr_2_	L2	46	>20/1

a Carried out under oxygen atmosphere: metal salt (0.02 mmol, 10 mol%), 1a (0.2 mmol), 2 mL MeOH.

b Isolated yields.

c dr was determined by ^1^H NMR.

d 4 mL methanol was used.

e Air atmosphere; nr: not reaction.

With the optimized reaction conditions in hand, we continued to investigate the substrate scope of the reaction (Table 2). Reactions of *N*-methyl substituted tryptamines that contain either a methyl or ethyl group at different positions of the indole ring proceeded smoothly to furnish the desired products 2a–e in good to excellent yields with moderate to excellent diastereoselectivities. Notably, the *N*-Bn and *N*-PMB substituted tryptamines were also suitable substrates for this reaction, the corresponding products 2f, **2**o were obtained in 70, 79% yields with >20/1 dr. *N*-Bn substituted substrates that contain different functional groups at different positions of the indole ring employed the reaction conditions well, affording the desired products 2g–m in good to excellent yields with high diastereoselectivities. Furthermore, the use of other alcohols, for instance, ethanol, *n*-butanol, *sec*-butyl alcohol or benzyl alcohol allowed the cyclic alkoxylation reaction smoothly (2p–s). However, when the steric and bulky *tert*-butyl alcohol was employed under the optimal conditions, no trace amount of desired product was observed. The applicability of this protocol was further demonstrated by the short, rapid construction of bio-active natural product CPC-1 in a total yield of 54% with 4/1 dr from material 1u (Table 2).

**Table 2:** Substrate scope of alkoxycyclization and concise synthesis of natural product CPC-1

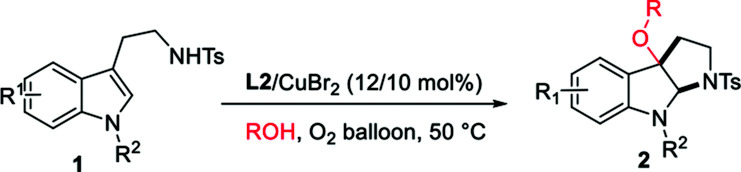
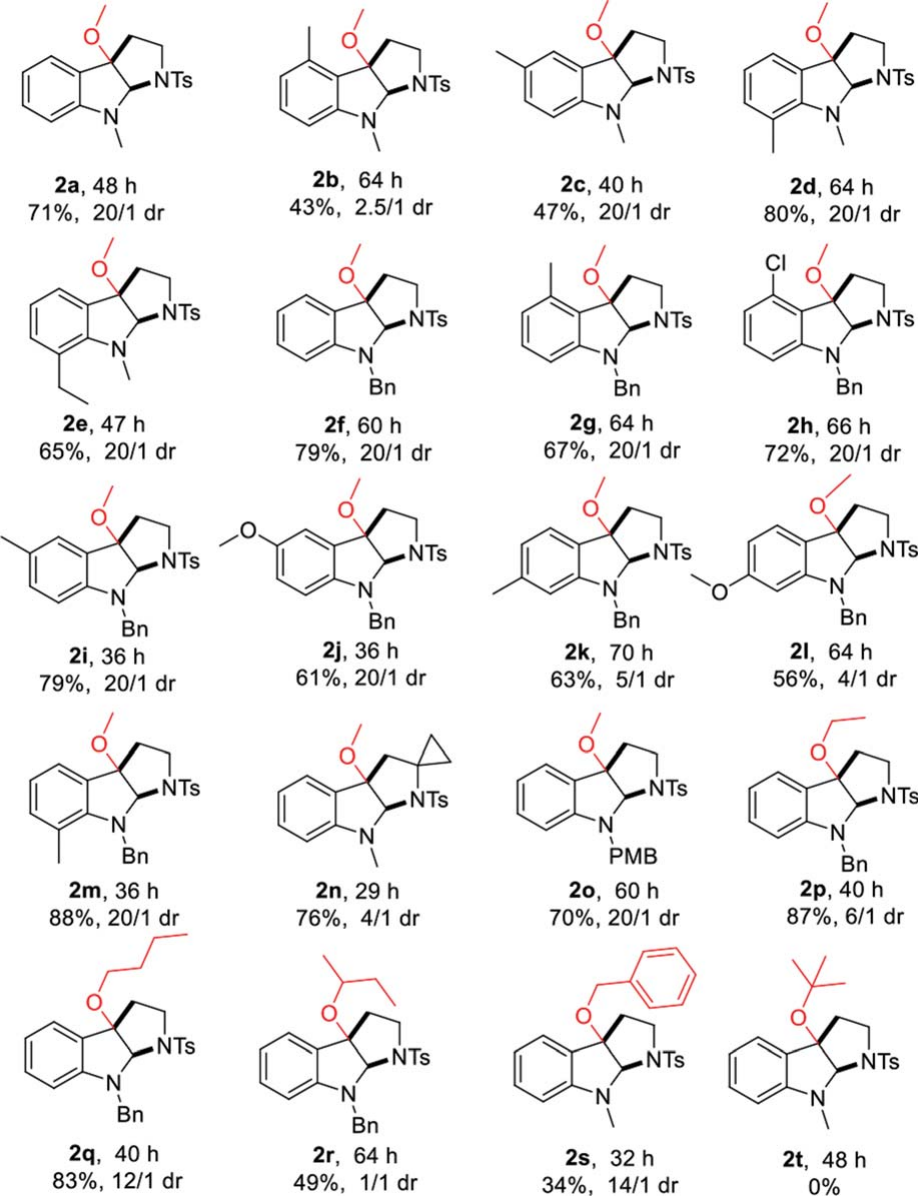
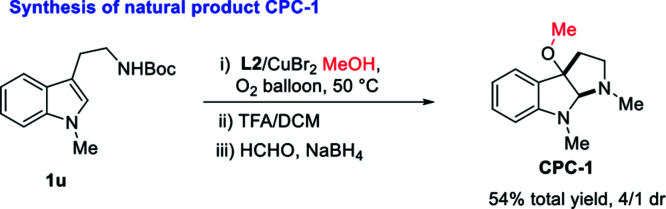

In order to gain insight into the mechanism of the methodology, several control experiments were carried out. As shown in Scheme 2, the radical scavenger, 2,2,6,6-tetramethylpiperidine1-oxyl (TEMPO), inhibited the alkoxycyclization process completely, suggesting that a radical process might be involved in this reaction (Scheme 2: eqn (1)).^13^ When the nucleophilic substrate 1-methyl indole was involved in the standard conditions (Scheme 2: eqn (2)), trace amount of 3-indole pyrrolidinoindoline adduct 4 was detected by HRMS, suggesting that the exposed carbocation intermediate may be the precursor for the formation of the 3-alkoxylation product. Besides, the amidyl radical addition process has been ruled out by the substrate scope investigation of 1n (Table 2) as the ring opening of cyclopropane moieties did not occur. When 1,3-dimethyl-indole (5) and *N*-4-dimethylbenzenesulfonamide (6) were involved in the standard conditions, the reaction did not take place (Scheme 2: eqn (3)), which indicated that this reaction proceeded *via* an intramolecular collaborative tandem process.

**Scheme 2:**
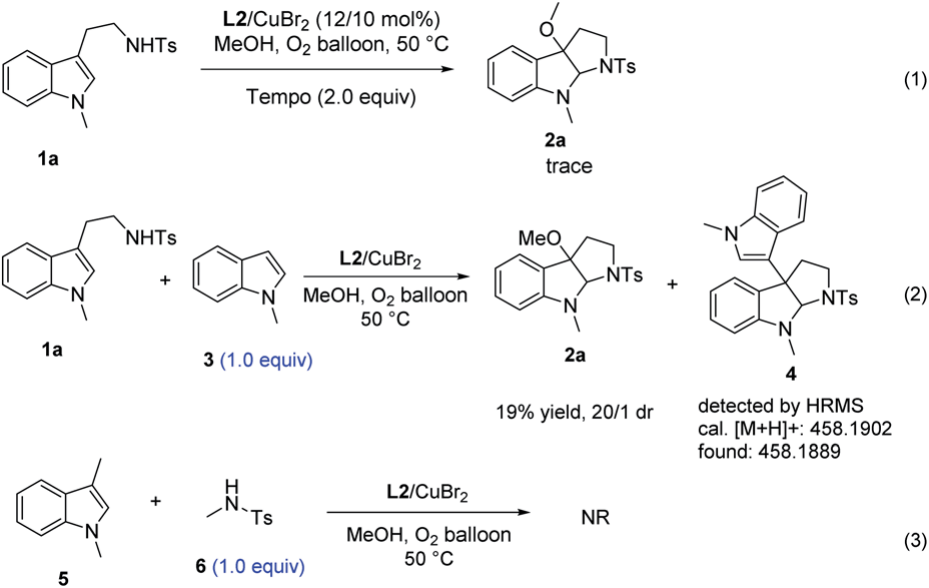
Control experiments.

Combining with the previous reports about copper-catalyzed carboamination,^10^ alkoxylation^12^ of alkene, a possible reaction pathway is proposed in Scheme 3. Initially, a ligand–exchange reaction of Cu(ii) species with substrate 1a proceeds to form the chelation intermediate A. Subsequent nitrogen intramolecular addition–cyclization forms the C3a Cu(ii) pyrrolidinoindoline intermediate B, Then, homolytic cleavage of carbon–Cu(ii) bond to generate the Cu(i) species and C3a radical intermediate C. The C3a radical could be oxidized by Cu^II^ species to generate the C3a cation intermediate D. Subsequent nucleophilic attack of alcohol delivers the product 2a. Meanwhile, Cu^II^ complex was produced *in situ* through the reaction of Ln–Cu^I^ complex with O_2_ on the basis of the previous reports,^14^ completing the catalytic cycle.

**Scheme 3:**
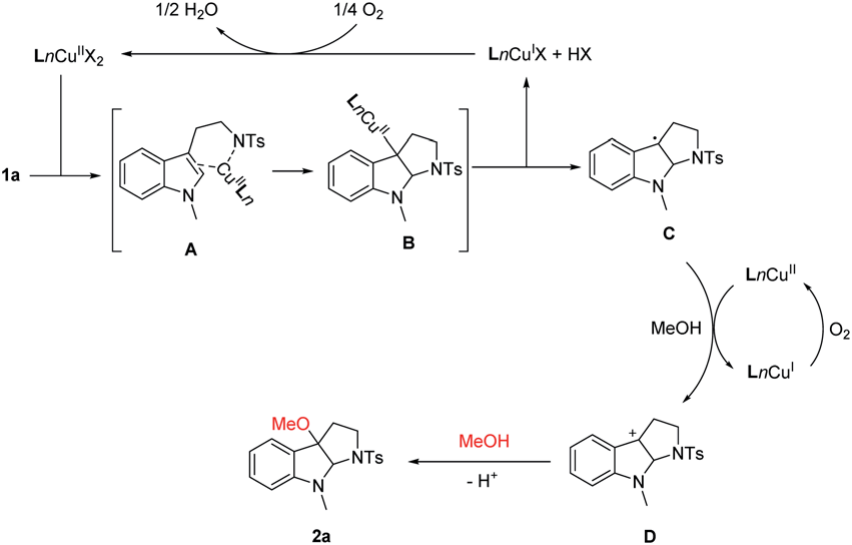
Plausible reaction pathway.

In conclusion, we have successfully developed copper-catalyzed alkoxycyclization of tryptamine under mild O_2_ oxidation conditions, affording C3a-alkoxylation pyrrolidinoindolines in good yields with high diastereoselectivities. This protocol was proved practicable and useful by the rapid concise total synthesis of natural product CPC-1. Mechanistic studies illustrated that the copper-catalyzed carboamination cyclization was favored to form the C-3 radical pyrrolidinoindoline intermediate, then a copper-catalyzed radical alkoxylation reaction proceeded to deliver the desired product. The extension of the present catalytic protocol to other useful reactions and biological evaluation of these products are undergoing in our laboratory.

## Conflicts of interest

There are no conflicts to declare.

## Supplementary Material

RA-011-D1RA02679H-s001
