# Supplementary material for: Copper-catalyzed aerobic oxidative radical alkoxycyclization of tryptamines to access 3-alkoxypyrroloindolines
Source: RSC Adv. 2021 May 19;11(29):18080–3. doi: 10.1039/d1ra02679h (PMC9033248; doi:10.1039/d1ra02679h)

Electronic Supplementary Material (ESI) for RSC Advances.
This journal is © The Royal Society of Chemistry 2021

Copper-catalyzed aerobic oxidative radical alkoxycyclization of tryptamines to access 3-alkoxypyrroloindolines

Wei Wang,[†] Jun-Rong Song,[†] Zhi-Yao Li, Ting Zhong, Qin Chi, Hai Ren* and Wei-Dong Pan*

School of Pharmaceutical Sciences, Guizhou University, Guizhou University, Huaxi Avenue South, Guiyang 550025, (China);
State Key Laboratory of Functions and Applications of Medicinal Plants, Guizhou Medical University, Guiyang 550014, (China); The Key Laboratory of Chemistry for Natural Products of Guizhou Province and Chinese Academy of Sciences, Guiyang 550014, (China).
E-mail: renh@gzcnp.cn; wdpan@163.com

Supporting Information

Content

1. **General information** 2
2. **Optimization of the reaction conditions**..3
3. **Mechanistic studies**..4
4. **General Procedure for Alkoxylation and Product Characterizations** 5
5. **Synthesis of partial substance**..18
6. **Synthesis of CPC-1 and Product Characterizations**......................................20
7. **^{1}H NMR and ^{13}C NMR Spectra of compounds**.......................................22

1. General information

Unless stated, otherwise all reactions were carried out under an atmosphere of nitrogen using standard Schlenk techniques. All solvents and reagents were obtained from commercial sources and were purified according to standard procedures before use. Column chromatography was performed on silica gel (Qingdao, 300 - 400 mesh) using the indicated eluents.NMR spectra were recorded on a Varian Mercury 400 MHz or Agilent Mercury 400 MHz spectrometer (^{1}H: 400 MHz, ^{13}C: 100 MHz) in chloroform-d or Agilent Mercury 600 MHz spectrometer (^{1}H: 600 MHz and ^{13}C: 150 MHz) in chloroform-d. ^{1}H and ^{13}C NMR spectra were internally referenced to the proton (^{1}H) of the internal TMS signal at 0.00 ppm or the solvent residue of DMSO at 2.54 ppm and the residual carbon nuclei (^{13}C) of the solvent at 77.0 or 40.5 ppm, respectively. Data for ^{1}H NMR were recorded as follows: chemical shift (δ, ppm), multiplicity (s = singlet, d = doublet, t = triplet, m = multiplet or unresolved, coupling constant(s) in Hz, integration). IR spectra were recorded using a FTIR spectrometer (IR 200) and the KBr disk method was adopted; High resolution mass spectra were obtained using Bruker ESI-QTOF mass spectrometry.

2. Optimization of the reaction conditions

2.1 Metal salt and volume of solvent screening[a]

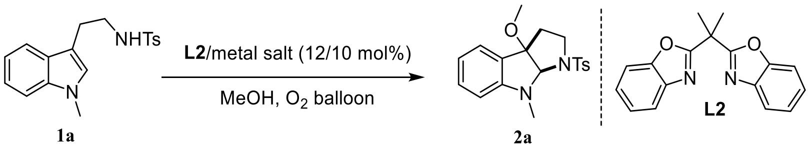

Entry	T (℃)	Metal salts	MeOH (mL)	*t* (h)	Yield (%)[b]	Dr[c]
1	30	$CuBr_2$	2	48	trace	-
2	50	$CuBr_2$	2	48	45	-
3	50	$CuBr_2$	4	42	71	>20/1
4	50	$CuBr_2$	8	48	49	-
5	70	$CuBr_2$	2	48	31	11/1

[a] Carried out under oxygen atmosphere: Metal salt (0.02 mmol, 10 mol%), **1a** (0.2 mmol); [b] Isolated yields. [c] dr was determined by ^{1}H-NMR of the crude product.

2.2 The influence of *N*-protecting group [a]

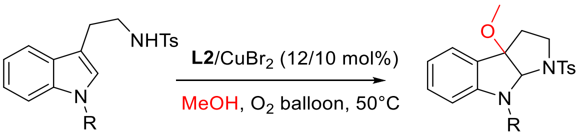

Entry	R	*t* (h)	Yield (%)	dr
1	Me	48	71	20/1
2	Bn	60	79	20/1
3	PMB	60	70	20/1
4	Boc	42	NR	-
5	Ac	48	NR	
6	Ts	48	NR	

[a] Carried out under oxygen atmosphere: Metal salt (0.02 mmol, 10 mol%), **1a** (0.2 mmol); [b]Yields were isolated yields.

2.3 Investigation of Cu (I) metal salts

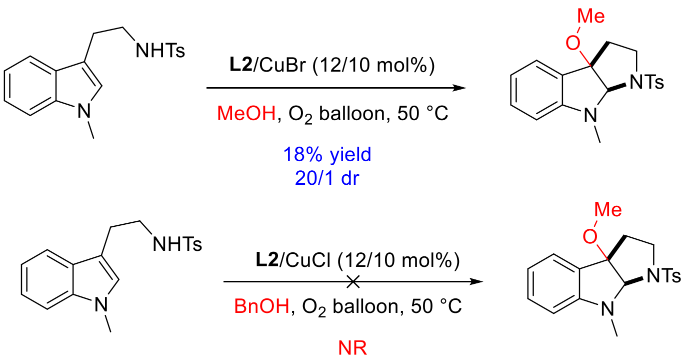

3. Mechanistic studies

3.1 Control experiments of 3-alkoxylation reaction

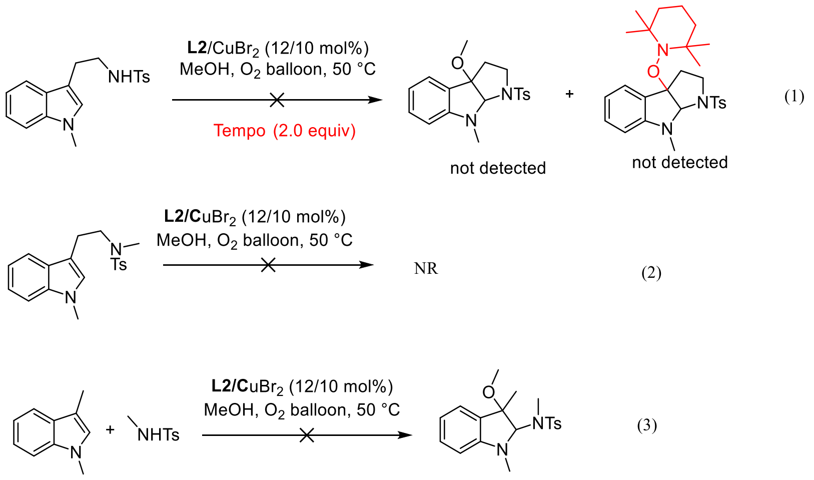

A 50 mL reaction tube was charged with $CuBr_2$ (4.46 mg, 0.02mmol, 10 mol%), tryptamine substrates (0.2 mmol, 1.0 equiv.), **L2** (6.67 mg, 0.024 mmol, 12 mol%) and 2,2,6,6-Tetramethyl-1-piperidinyloxy (TEMPO) (0.4 mmol, 2.0 equiv) before O_2 replacement operation, then alcohol (4mL) was added. The reaction mixturewas stirred vigorously at 50 ˚C and monitored by TLC.

4. General Procedure for Alkoxylation and Product Characterizations

4.1 Synthesis of alkoxypyrroloindolines

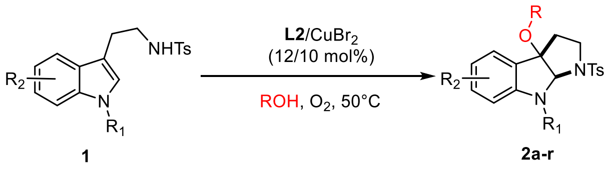

A 50 mL reaction tube was charged with $CuBr_2$ (4.46 mg, 0.02 mmol, 10 mol%), tryptamine substrates (0.2 mmol, 1.0 equiv) and ligand (0.024 mmol, 12 mol%) before O_2 replacement operation, then alcohol (4 mL) was added. Then the reaction mixture was heat to 50 ℃. After the reaction completed, the reaction mixture was filtered with short silica gel column, concentrated under reduced pressure and the residue was purified by chromatography on silica gel (PE/EA, 10:1) to afford alkoxypyrroloindoline.

4.2 Scale experiments

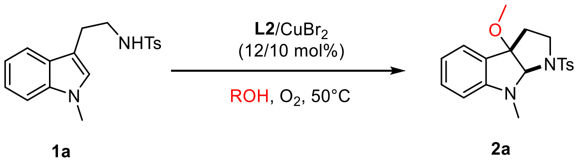

A 200 mL reaction tube was charged with $CuBr_2$ (89.4 mg, 0.4 mmol, 10 mol%), tryptamine substrates **1a** (1.31 g, 4 mmol, 1.0 equiv) and ligand (133.6 mg, 0.48 mmol, 12 mol%) before O_2 replacement operation, then alcohol (80 mL) was added. Then the reaction mixture was heat to 50 ℃. After 72 h, the reaction mixture was filtered with short silica gel column, concentrated under reduced pressure and the residue was purified by chromatography on silica gel (PE/EA, 10:1) to afford alkoxypyrroloindoline **2a** 0.813 g.

4.3 Product Characterizations

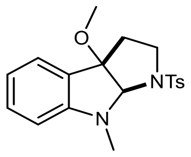

2a

2a White solid, 51.0 mg, 71% yield, 20/1 dr.

For the mixture of the two diastereomers: ^{1}**H NMR** (600 MHz, Chloroform-*d*) *δ* 7.79 - 7.76 (m, 2 + 0.32 H), 7.34 - 7.33 (m, 2 + 0.32 H), 7.28 (dd, *J* = 12.0, 6.0 Hz, 0.16 H), 7.22 (td, *J* = 7.8, 1.2 Hz, 1 H), 7.15 (d, *J* = 6.0 Hz, 0.16 H), 7.06 (d, *J* = 6.0 Hz, 1 H), 6.71 (td, *J* = 7.8, 1.2 Hz, 1 H), 6.47 (d, *J* = 12.0 Hz, 1 H), 6.34(d, *J* = 6.0 Hz, 0.16 H), 5.36 (s, 1 H), 5.36 (s, 0.16 H), 3.58 - 3.55 (m, 1 + 0.16 H), 3.12 - 3.08 (m, 1 + 0.16 H), 3.02 (s, 3 H), 2.99 (s, 0.48 H), 2.97 (s, 0.48 H), 2.97 (s, 3 H), 2.44 - 2.44 (m, 3 + 0.48 H), 2.09 - 2.04 (m, 1 + 0.16 H), 1.84 - 1.79 (m, 1 + 0.16 H);

^{13}**C NMR** (150 MHz, Chloroform-*d*) *δ* 151.9, 143.7, 136.2, 130.6, 129.7, 127.3, 124.8, 123.9, 117.8, 106.7, 93.7, 86.1, 52.7, 47.4, 38.6, 31.4, 21.5;

HRMS-ESI: Exact mass calcd. for $C_{19}H_{23}O_{3}N_{2}S$ $[M+H]^{+}$: 359.1424; Found: 359.1421;

IR (KBr): 2880.75, 2358.79, 1612.40, 1489.14, 1342.07, 1276.23, 1162.10, 1101.82, 1072.04, 1028.07, 1014.24, 931.01, 819.27 ,735.84, 609.51, 545.47.

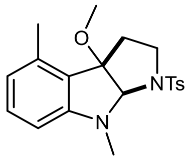

2b

2b White solid, 31.7 mg, 43% yield, 2.5/1 dr;

For the mixture of the two diastereomers: ^{1}**H NMR** (600 MHz, Chloroform-*d*) *δ* 7.80 - 2.78 (m, 2 + 0.74 H), 7.34 (d, *J* = 6.0 Hz, 2 + 0.74 H), 7.10 (t, *J* = 6.0 Hz, 1 H), 6.48 (d, *J* = 6.0 Hz, 1 H), 6.30 (d, *J* = 6.0 Hz, 1 H), 6.19 (d, *J* = 12.0 Hz, 0.37 H), 5.37 (s, 0.37 H), 5.37 (s, 1 H), 3.59 - 3.55 (m, 1 + 0.37 H), 3.22 - 3.18 (m, 1 + 0.37 H), 2.99 (s, 3 H), 2.97 (s, 1.11 H),2.92 (s, 3 + 1.11 H), 2.44 - 2.45 (m, 3+ 1.11 H), 2.24 (s, 1.11 H),

2.20 (s, 3 H), 2.18 - 2.15 (m, 1 H), 2.14 - 2.11 (m, 0.37 H),1.89 - 1.84 (m, 1 + 0.37 H);

^{13}C NMR (150 MHz, Chloroform-*d*) *δ* 151.8, 150.9, 143.8, 143.7, 136.3, 135.1, 134.7, 134.0, 130.4, 129.8, 129.7, 127.4, 121.9, 120.0, 105.8, 104.3, 94.8, 86.3, 52.8, 52.6, 47.4, 47.3, 38.3, 38.0, 31.7, 31.5, 21.6, 17.2, 17.0;

HRMS-ESI: Exact mass calcd. for $C_{20}H_{25}O_3N_2S$ $[M+H]^+$: 373.1580; Found: 373.1578;

IR (KBr): 2979.44, 1598.24, 1472.99, 1340.75, 1281.00, 1234.87, 1161.33, 1094.36, 1074.00, 1014.25, 943.90, 873.57, 818.52, 664.29, 599.49.

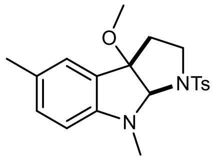

2c

2c White solid, 34.9 mg, 47% yield, 20/1 dr;

^{1}H NMR (600 MHz, Chloroform-*d*) *δ* 7.78 (d, *J* = 12.0 Hz, 2 H), 7.33 (d, *J* = 12.0 Hz, 2 H), 7.02 (d, *J* = 6.0 Hz, 1 H), 6.87 (s, 1 H), 6.39 (d, *J* = 6.0 Hz, 1 H), 5.32 (s, 1 H), 3.57 - 3.53 (m, 1 H), 3.13 -3.09 (m, 1 H), 2.99 (s, 3 H), 2.92 (s, 3 H), 2.44 (s, 3 H), 2.25 (s, 3 H), 2.08 - 2.04 (m, 1 H), 1.85 - 1.80 (m, 1 H);

^{13}C NMR (150 MHz, Chloroform-*d*) *δ* 149.9, 143.6, 136.3, 131.0, 129.7, 127.3, 127.2, 125.0, 124.4, 106.9, 93.8, 86.5, 52.7, 47.5, 38.5, 32.0, 21.5, 20.7;

HRMS-ESI: Exact mass calcd. for $C_{20}H_{25}O_3N_2S$ $[M+H]^+$: 373.1580; Found: 373.1578;

IR (KBr): 2884.84, 2358.58, 1621.28, 1497.65, 1341.77, 1288.09, 1163.25, 1102.67, 1071.64, 1029.72, 1014.90, 936.22, 818.49, 674.85, 662.16, 599.34.

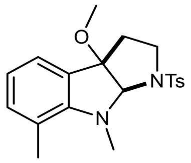

2d

2d White solid, 60.0 mg, 80% yield, 20/1 dr;

^{1}H NMR (600 MHz, Chloroform-*d*) *δ* 7.78 (d, *J* = 12.0 Hz, 2 H), 7.29 - 7.26 (m, 2 H), 6.98 - 6.97 (m, 2 H), 6.77 (t, *J* = 7.4 Hz, 1 H), 5.20 (s, 1 H), 3.61 - 3.58 (m, 1 H), 3.14 (s, 3 H), 2.99 (s, 3 H), 2.92 - 2.88 (m, 1 H), 2.41 (s, 3 H), 2.21 (s, 3 H), 2.19 - 2.16 (m, 2 H);

^{13}C NMR (150 MHz, Chloroform-*d*) *δ* 151.3, 143.2, 137.1, 133.3, 129.4, 127.3, 127.3, 121.9, 121.7, 120.6, 93.3, 88.6, 52.8, 46.4, 38.3, 38.1, 21.4, 18.8;

HRMS-ESI: Exact mass calcd. for $C_{20}H_{25}O_3N_2S$ $[M+H]^+$: 373.1580; Found: 373.1579;

IR (KBr): 2907.15, 1604.08, 1465.41, 1411.50, 1237.62, 1092.30, 944.15, 851.78, 836.64, 813.72, 784.69, 752.36, 734.77, 708.41, 573.90.

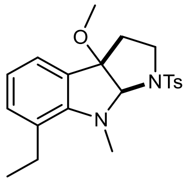

2e

2e White solid, 50.0 mg, 65% yield, 20/1 dr;

For the mixture of the two diastereomers: **^{1}H NMR** (600 MHz, Chloroform-*d*) *δ* 7.78 - 7.76 (m, 2 + 0.26 H), 7.28 - 7.26 (m, 2 + 0.26 H), 7.13 (d, *J* = 1.8 Hz, 0.13 H), 7.06 (d, *J* = 1.8 Hz, 0.13 H), 7.03 (d, *J* = 7.4 Hz, 1H), 6.98 (d, *J* =6.0 Hz, 1H), 6.83 (t, *J* = 6.0 Hz, 1 H), 5.21 (s, 1 H), 5.20 (s, 0.13 H), 3.63 - 3.59 (m, 1 + 0.13 H), 3.12 (s, 3 H), 3.11 (s, 0.39 H), 3.01 (s, 0.39 H), 3.00 (s, 3H), 2.92 - 2.88 (m, 1 + 0.13 H), 2.56 - 2.51 (m, 2 + 0.26 H), 3.41 (s, 0.39 H), 2.40 (s, 3 H), 2.22 -2.17 (m, 2 + 0.26 H), 1.12 - 1.10 (m, 3 + 0.39 H);

^{13}C NMR (150 MHz, Chloroform-*d*) *δ* 150.8, 143.1, 137.3, 131.2, 129.4, 128.2, 127.6, 127.2, 121.8, 120.8, 93.3, 88.8, 52.9, 46.3, 38.9, 38.2, 24.5, 21.4, 14.3;

HRMS-ESI: Exact mass calcd. for $C_{21}H_{27}O_3N_2S$ $[M+H]^+$: 387.1737; Found: 387.1735;

IR (KBr): 2964.05, 1600.63, 1454.82, 1340.92, 1159.35, 1118.51, 1079.94, 944.08, 812.27, 752.10, 707.52, 687.68, 659.60, 580.25, 546.82.

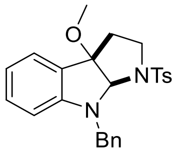

2f

2f White solid, 68.7 mg, 79% yield, 20/1 dr;

^{1}H NMR (600 MHz, Chloroform-*d*) *δ* 7. 21 (d, *J* = 6.0 Hz, 2 H), 7.37 (d, *J* = 12.0 Hz, 2 H), 7.31 - 7.28 (m, 4 H), 7.25 - 7.23 (m, 1 H), 7.15 (t, *J* = 6.0 Hz, 1 H), 7.07 (d, *J* = 6.0 Hz, 1 H), 6.71 (t, *J* = 6.0 Hz, 1 H), 6.47 (d, *J* = 6.0 Hz, 1 H), 5.54 (s, 1 H), 4.69 (ABd, *J* = 24.0, 12.0 Hz, 2 H), 3.64 - 3.60 (m, 1 H), 3.20 - 3.15 (td, *J* = 12.0, 6.0 Hz, 1 H), 2.82 (s, 3 H), 2.42 (s, 3 H), 2.10 - 2.07 (m, 1 H), 1.81 - 1.75 (m, 1 H).

HRMS-ESI: Exact mass calcd. for $C_{25}H_{27}O_3N_2S$ $[M+H]^+$: 435.1737; Found: 435.1734. All analytical data are consistent with literature (*Org.Biomol. Chem.* 2020, **18**, 32-35).

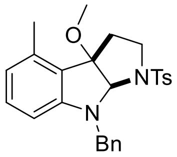

2g

2g yellow liquid, 60.0 mg, 67% yield, 20/1 dr;

^{1}H NMR (600 MHz, Chloroform-*d*) *δ* 7.71 (d, *J* = 12.0 Hz, 2 H), 7.37 (d, *J* = 6.0 Hz, 2 H), 7.30 - 7.22 (m, 5 H), 7.05 (t, *J* = 6.0 Hz, 1 H), 6.48 (d, *J* = 6.0 Hz, 1 H), 6.34 (d, *J* = 6.0 Hz, 1 H), 5.56 (s, 1 H), 4.69 (ABd, *J* = 18.0, 12.0 Hz, 2 H), 3.65 - 3.61 (m, 1 H), 3.27 - 3.22 (m, 1 H), 2.77 (s, 3 H), 2.41 (s, 3 H), 2.23 - 2.18 (m, 4 H), 1.84 - 1.79 (m, 1 H);

^{13}C NMR (150 MHz, Chloroform-*d*) *δ* 151.1, 143.6, 138.1, 136.3, 135.5, 130.4, 129.7, 128.4, 127.6, 127.3, 127.0, 121.7, 119.9, 104.7, 95.0, 84.4, 52.5, 48.1, 47.1, 38.5, 21.5, 17.0;

HRMS-ESI: Exact mass calcd. for $C_{26}H_{29}O_3N_2S$ $[M+H]^+$: 449.1893; Found: 449.1892;

IR (KBr): 2358.54, 1653.13, 1595.80, 1455.25, 1344.46, 1157.41, 1091.55, 927.47, 811.25, 764.71, 743.95, 704.46, 658.85, 582.11, 543.61.

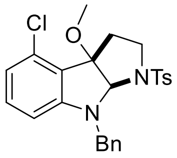

2h

2h white solid, 67.4 mg, 72% yield, >20/1 dr.

^{1}H NMR (600 MHz, Chloroform-*d*) *δ* 7.72 (d, *J* = 12.0 Hz, 2 H), 7.37 (d, *J* = 6.0 Hz, 2 H), 7.33 - 7.25 (m, 5 H), 7.08 (t, *J* = 6.0 Hz, 1 H), 6.63 (d, *J* = 6.0 Hz, 1 H), 6.38 (d, *J* = 6.0 Hz, 1 H), 5.56 (s, 1 H), 4.69 (ABd, *J* = 18.0, 12.0 Hz, 2 H), 3.65 (dd, *J* = 12.0, 6.0 Hz, 1 H), 3.23 - 3.18 (m, 1 H), 2.85 (s, 3 H), 2.46 - 2.42 (m, 4 H), 1.74 - 1.69 (m, 1 H);

^{13}C NMR (150 MHz, Chloroform-*d*) *δ* 152.6, 143.9, 137.6, 136.1, 131.9, 131.5, 129.8, 128.6, 127.5, 127.3, 127.3, 120.4, 118.5, 105.3, 94.7, 84.6, 53.0, 47.9, 47.1, 37.7, 21.6;

HRMS-ESI: Exact mass calcd. for $C_{25}H_{26}O_3N_2ClS$ $[M+H]^+$: 469.1347; Found: 469.1348;

IR (KBr): 2925.38, 1596.26, 1493.43, 1330.72, 1236.96, 1005.30, 925.74, 856.22, 811.51, 757.62, 704.35, 670.11, 574.04, 543.72.

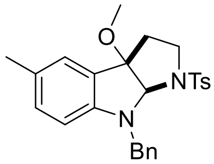

2i

2i yellow liquid, 70.7 mg, 79% yield, 20/1 dr;

^{1}H NMR (600 MHz, Chloroform-*d*)*δ* 7.72 (d, *J* = 6.0 Hz, 2 H), 7.37 (d, *J* = 6.0 Hz, 2 H), 7.30 - 7.22 (m, 5 H), 6.96 (d, *J* = 6.0 Hz, 1 H), 6.88 (s, 1 H), 6.37 (d, *J* = 6.0 Hz, 1 H), 5.52 (s, 1 H), 4.66 (ABd, *J* = 12.0, 6.0 Hz, 2 H), 3.62 - 3.59 (m, 1 H), 3.20 - 3.15 (m, 1 H), 2.81 (s, 3 H), 2.42 (s, 3 H), 2.24 (s, 3 H), 2.08 - 2.05 (m, 1 H), 1.81 - 1.76 (m, 1 H);

^{13}C NMR (150 MHz, Chloroform-*d*) *δ* 149.1, 143.6, 138.3, 136.4, 131.0, 129.7, 128.4, 127.6, 127.3, 127.2, 127.0, 124.7, 107.1, 94.1, 84.6, 52.6, 48.4, 47.3, 39.3, 21.5, 20.7;

HRMS-ESI: Exact mass calcd. for $C_{26}H_{28}O_3N_2NaS$ $[M+Na]^+$: 471.1713; Found: 471.1707;

IR (KBr): 2918.56, 1496.48, 1453.59, 1347.55, 1288.79, 1166.58, 1117.59, 1068.30, 1028.90, 807.79, 744.49, 701.32, 663.39, 582.09, 546.93.

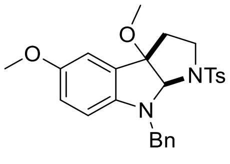

2j

2j yellow liquid, 56.2 mg, 61% yield, 20/1 dr;

^{1}H NMR (600 MHz, Chloroform-*d*) *δ* 7.72 (d, *J* = 6.0 Hz, 2 H), 7.37 (d, *J* = 6.0 Hz, 2 H), 7.30 -7.23 (m, 5 H), 6.73 (dd, *J* = 12.0, 6.0 Hz, 1 H), 6.69 (d, *J* = 3.0 Hz, 1 H), 6.37 (d, *J* = 6.0 Hz, 1 H), 5.53 (s, 1 H), 4.64 (ABd, *J* = 22.0, 15.0 Hz, 2 H), 3.72 (s, 3 H), 3.63 - 3.59 (m, 1 H), 3.22 - 3.17 (m, 1 H), 2.81 (s, 3 H), 2.42 (s, 3 H), 2.10 - 2.06 (m, 1 H), 1.84 - 1.78 (m, 1 H);

^{13}C NMR (150 MHz, Chloroform-*d*) 152.8, 145.4, 143.6, 138.3, 136.4, 129.7, 128.4, 127.6, 127.3, 127.0, 126.1, 115.8, 110.5, 108.1, 94.1, 84.9, 56.0, 52.7, 49.1, 47.3, 39.4, 21.5;

HRMS-ESI: Exact mass calcd. for $C_{26}H_{29}O_3N_2S$ $[M+H]^+$: 465.1843; Found: 465.1843;

IR (KBr): 2882.38, 1492.03, 1338.51, 1273.37, 1157.22, 1037.03, 982.62, 939.48, 878.32, 804.29, 757.53, 667.19, 574.71 22.48.

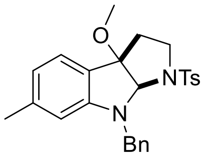

2k

2k yellow liquid, 56.2 mg, 63% yield, 5/1 dr;

^{1}H NMR (600 MHz, Chloroform-*d*) *δ* 7.71 (d, *J* = 6.0 Hz, 2 H), 7.38 (d, *J* = 6.0 Hz, 2 H), 7.31 - 7.23 (m, 5 H), 6.96 (d, *J* = 6.0 Hz, 1 H), 6.54 (d, *J* = 6.0 Hz, 1 H), 6.31 (s, 1 H), 5.52 (s, 1 H), 4.67 (ABd, *J* = 12.0, 6.0 Hz, 2 H), 3.63 - 3.59 (m, 1 H), 3.19 - 3.14

(m, 1 H), 2.82 (s, 3 H), 2.41 (s, 3 H), 2.26 (s, 3 H), 2.07 - 2.04 (m, 1 H), 1.77 - 1.72 (m, 1 H);

^{13}C NMR (150 MHz, Chloroform-*d*) *δ* 151.5, 143.6, 140.9, 138.3, 136.4, 129.7, 128.4, 127.5, 127.3, 127.0, 124.1, 121.7, 118.6, 107.6, 93.9, 84.6, 52.6, 47.8, 47.3, 39.4, 21.9, 21.5;

HRMS-ESI: Exact mass calcd. for $C_{26}H_{29}O_3N_2S$ $[M+H]^+$: 449.1893; Found: 449.1893;

IR (KBr): 2827.13, 1615.20, 1494.88, 1453.06, 1346.71, 1159.88, 1072.47, 933.36, 807.37, 743.79, 709.21, 661.59, 600.84, 566.55, 544.12.

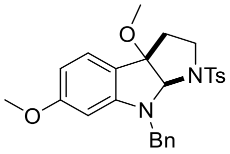

2l

2l yellow liquid, 56.1 mg, 56% yield, 4/1 dr;

For the mixture of the two diastereomers: **^{1}H NMR** (600 MHz, Chloroform-*d*) *δ* 7.72 - 7.70(m, 2 + 0.5 H), 7.39 - 7.37 (m, 2 + 0.5 H), 7.32 - 7.23 (m, 5 + 1.75 H), 6.96 (d, *J* = 12.0 Hz, 1 H), 6.24 (dd, *J* = 6.0, 3.0 Hz, 1 H), 6.03 (s, 1 H), 6.02 (s, 0.25 H), 5.52 (s, 1 H), 5.51 (s, 0.25 H), 4.73 - 4.61 (m, 2 + 0.5 H), 3.74 (s, 0.75 H), 3.71 (s, 3 H), 3.63 - 3.60 (m, 1 + 0.25 H), 3.18 - 3.13 (m, 1 + 0.25 H),2.85 (s, 0.75 H), 2.83 (s, 3 H), 2.43 (s, 0.75 H), 2.42 (s, 3 H), 2.05 - 2.02 (m, 1 + 0.25 H), 1.74 - 1.68 (m, 1 + 0.25 H);

^{13}C NMR (150 MHz, Chloroform-*d*) *δ* 162.5, 152.8, 143.7, 138.1, 136.4, 129.8, 128.5, 127.6, 127.3, 127.1, 125.0, 116.9, 102.8, 93.7, 93.6, 84.9, 55.3, 52.5, 47.9, 47.3, 39.4, 21.5;

HRMS-ESI: Exact mass calcd. for $C_{26}H_{29}O_4N_2S$ $[M+H]^+$: 465.1843; Found: 465.1843;

IR (KBr): 2880.46, 1612.19, 1489.16, 1430.79, 1342.02, 1161.81, 1101.52, 1071.75, 1027.78, 841.22, 818.98, 735.51, 574.15, 545.13.

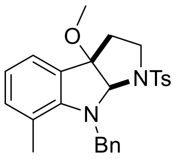

2m

2m yellow liquid, 81.1 mg, 88% yield, >20/1 dr;

^{1}H NMR (600 MHz, Chloroform-*d*) *δ* 7.72 (d, *J* = 12.0 Hz, 2 H), 7.28 - 7.22 (m, 7 H), 7.00 (d, *J* = 6.0 Hz, 1 H), 6.93 (d, *J* = 6.0 Hz, 1 H), 6.74 (t, *J* =6.0 Hz, 1 H), 5.43 (s, 1 H), 4.98 (d, *J* = 18.0 Hz, 1 H),4.74 (d, *J* = 12.0 Hz, 1 H), 3.61 - 3.57 (m, 1 H), 3.09 - 3.04 (m, 1 H), 2.50 (s, 3 H), 2.41 (s, 3 H), 2.36 (s, 3 H), 2.04 - 2.01 (m, 1 H), 1.91 - 1.87 (m, 1 H);

^{13}C NMR (150 MHz, Chloroform-*d*) *δ* 149.1, 143.4, 138.7, 136.8, 133.7, 129.6, 128.5, 128.1, 127.2, 126.8, 122.4, 119.6, 119.4, 93.2, 84.3, 52.1, 50.6, 46.6, 39.5, 21.5, 19.6;

HRMS-ESI: Exact mass calcd. for $C_{26}H_{29}O_3N_2S$ $[M+H]^+$: 449.1893; Found: 449.1891;

IR(KBr): 2937.15, 1602.29, 1451.44, 1422.90, 1383.56, 1343.02, 1301.19, 1255.38, 1153.58, 945.69, 807.65, 744.32, 696.87, 581.36, 543.11.

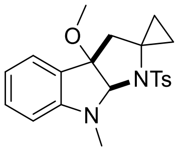

2n

2n yellow liquid, 29.2 mg, 76% yield, 4/1 dr;

For the mixture of the two diastereomers: **^{1}H NMR** (600 MHz, Chloroform-*d*) *δ* 7.83 - 7.80 (m, 2 + 0.42 H), 7.35 (d, *J* = 6.0 Hz, 2 + 0.42 H), 7.29 (dd, *J* = 6.0, 2.64 Hz, 0.21 H), 7.20 (td, *J* = 12.0, 6.0 Hz, 1 H), 7.08 (d, *J* = 6.0 Hz, 0.21 H), 7.01 (d, *J* = 6.0 Hz, 1 H), 6.66 (t,*J* = 18.0, 6.0 Hz, 1 H), 6.43 (d, *J* = 12.0 Hz, 1 H), 6.30 (d, *J* = 6.0 Hz, 0.21 H), 5.66 (s, 1 H), 5.65 (s, 0.21 H), 3.05 (s, 0.63 H), 3.04 (s, 3 H), 2.97 (s, 3 H), 2.95 (s, 0.63 H),2.46 (s, 3 + 0.63 H), 1.95 - 1.91(m, 1 + 0.21 H), 1.79 - 1.77 (m, 1 + 0.21 H), 1.40 - 1.38 (m, 1 + 0.21 H), 0.57 - 0.53 (m, 1 + 0.21 H), 0.37 - 0.33 (m, 1 + 0.21 H), 0.15 - 0.12 (m, 0.21 H), 0.19 - 0.22 (m, 1 H);

^{13}C NMR (150 MHz, Chloroform-*d*) *δ* 152.5, 151.5, 143.9, 143.8, 137.0, 136.8, 133.2, 130.5, 129.8, 129.7, 127.8, 127.8, 126.9, 126.2, 124.0, 117.2, 108.5, 107.1, 105.6, 92.4, 92.1, 88.1, 88.0, 52.6, 52.5, 46.8, 46.7, 41.8, 30.0, 29.7, 21.6, 14.5, 5.8;

HRMS-ESI: Exact mass calcd. for $C_{21}H_{25}O_3N_2S$ $[M+H]^+$: 385.1580; Found: 385.1579;

IR (KBr): 2928.29, 1609.97, 1492.70, 1351.52, 1165.27, 1092.20, 1041.62, 1006.66, 913.52, 812.99, 724.58, 662.07, 549.52.

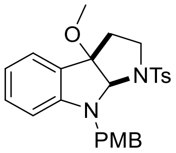

2o

2o White solid, 65.2 mg, 70% yield, 20/1 dr；

^{1}H NMR (600 MHz, Chloroform-*d*) *δ* 7.72 (d, *J* = 6.0 Hz, 2 H), 7.32 - 7.29 (m, 4 H), 7.15 (t, *J* = 6.0 Hz, 1H), 7.06 (d, *J* = 6.0 Hz, 1 H), 6.84 (d, *J* = 6.0 Hz, 2 H), 6.70 (t, *J* = 6.0 Hz, 1 H), 6.51 (d, *J* = 6.0 Hz, 1 H), 5.52 (s, 1 H), 4.62 (s, 2 H), 3.78 (s, 3 H), 3.60-3.63 (m, 1 H), 3.19 -3.14 (m, 1 H), 2.78 (s, 3 H), 2.42 (s, 3 H), 2.09-2.04 (m, 1 H), 1.80-1.74 (m, 1 H)；

^{13}C NMR (150 MHz, Chloroform-*d*) *δ* 158.7, 151.2, 143.7, 136.4, 130.5, 130.1, 129.7, 128.9, 127.3, 124.7, 124.3, 117.7, 113.8, 107.0, 94.0, 84.1, 55.2, 52.6, 47.4, 47.2, 39.5, 21.5；

HRMS-ESI: Exact mass calcd. for $C_{26}H_{29}O_4N_2S$ $[M+H]^+$: 465.1843; Found: 765.1843;

IR (KBr): 2932.38, 2358.87, 1733.91, 1609.39, 1512.36, 1488.34, 1346.85, 1245.74.

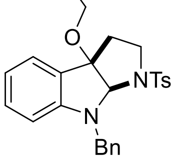

2p

2p yellow solid, 77.8 mg, 87% yield, 6/1 dr;

^{1}H NMR (600 MHz, Chloroform-*d*) δ 7.72 (d, *J* = 12.0 Hz, 2 H), 7.35 (d, *J* = 6.0 Hz, 2 H), 7.32 - 7.25 (m, 5 H), 7.15 - 7.12 (m, 1 H), 7.07 (d, *J* = 12.0 Hz, 1 H), 6.69 (t, *J* = 6.0 Hz, 1 H), 6.45 (d, *J* = 12.0 Hz, 1 H), 5.53 (s, 1 H), 4.68 (s, 2 H),3.66 - 3.62 (m, 1 H), 3.21 - 3.16 (m, 1 H), 3.05 - 2.99 (m, 1 H), 2.85 - 2.80 (m, 1 H), 2.43 (s, 3 H), 2.10 - 2.06 (m, 1 H), 1.81 - 1.76 (m, 1 H), 0.94 (t, 6.0 Hz, 3 H);

^{13}C NMR (150 MHz, Chloroform-*d*) δ 150.9, 143.7, 138.2, 136.3, 130.4, 129.7, 128.5, 127.5, 127.3, 127.1, 125.6, 124.1, 117.8, 107.0, 93.6, 84.9, 60.6, 48.1, 47.2, 39.8, 21.5, 15.4;

HRMS-ESI: Exact mass calcd. for $C_{26}H_{29}O_3N_2S$ $[M+H]^+$: 449.1893; Found: 449.1892;

IR (KBr): 2979.81, 1609.32, 1492.74, 1454.41, 1344.71, 1328.59, 1294.23, 1160.55, 1101.10, 1052.40, 939.41, 746.44, 729.94, 671.28 ,571.19.

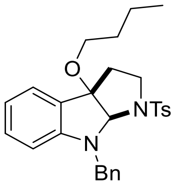

2q

2q yellow liquid, 79.0 mg, 83% yield, 12/1 dr;

^{1}H NMR (600 MHz, Chloroform-*d*) δ 7.72 (d, *J* = 6.0 Hz, 2 H), 7.38 (d, *J* = 6.0 Hz, 2 H), 7.31 - 7.24 (m, 5 H), 7.14 (t, *J* = 6.0 Hz, 1 H), 7.06 (d, *J* = 6.0 Hz, 1 H), 6.69 (t, *J* = 6.0 Hz, 1 H), 6.47 (d, *J* = 12.0 Hz, 1 H), 5.48 (s, 1 H), 4.69 (ABd, *J* = 18.0, 24.0 Hz, 2 H), 3.66 - 3.62 (m, 1 H), 3.20 - 3.15 (m, 1 H), 2.95 - 2.91 (m, 1 H), 2.73 - 2.69 (m, 1 H), 2.41 (s, 3 H), 2.07 - 2.05 (m, 1 H), 1.79 - 1.74 (m, 1 H), 1.28 - 1.23 (m, 2 H), 1.17 - 1.11 (m, 2 H), 0.80 (t, *J* = 6.0 Hz, 3 H);

^{13}C NMR (150 MHz, Chloroform-*d*) δ 151.0, 143.6, 138.2, 136.4, 130.4, 129.7, 128.4, 127.6, 127.3, 127.1, 125.6, 124.2, 117.7, 107.0, 93.5, 84.7, 64.8, 48.0, 47.3, 39.6, 31.9, 21.5, 19.2, 13.8;

HRMS-ESI: Exact mass calcd. for $C_{28}H_{33}O_3N_2S$ $[M+H]^+$: 477.2206; Found: 477.2212;

IR (KBr): 3137.42, 1608.94, 1401.44, 1161.39, 1082.63, 741.62, 661.36, 543.48.

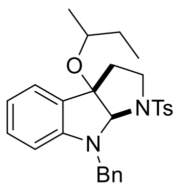

2r

2r yellow liquid, 46.2 mg, 49% yield, 1/1 dr;

For the mixture of the two diastereomers: **^{1}H NMR** (600 MHz, Chloroform-*d*) *δ* 7.75 - 7.73 (m, 2 + 1.14 H), 7.41 - 7.39 (m, 2 + 1.14 H), 7.32 - 7.25 (m, 5 + 2.85 H), 7.16 - 7.08 (m, 2 + 1.14 H), 6.70 - 6.68 (m, 1 + 0.57 H), 6.50 - 6.48 (m, 1 + 0.57 H), 5.47 (s, 0.57 H), 5.45 (s, 1 H), 4.79 - 4.74 (m, 1 + 0.57 H), 4.64 - 4.60 (m, 1 + 0.57 H), 3.63 - 3.60 (m, 1 + 0.57 H), 3.06-3.00 (m, 2 + 1.14 H), 2.43 - 2.42 (m, 3 + 1.71 H), 2.07 - 2.04 (m, 1 + 0.57H), 1.73 - 1.71 (m, 1 + 0.57H), 1.21 - 1.17 (m, 2 + 1.14H), 0.78 - 0.76 (m, 3 + 1.71H), 0.64 -0.59 (m, 3 + 1.71H);

^{13}C NMR (150 MHz, Chloroform-*d*) *δ* 151.3, 143.7, 138.4, 138.3, 136.6, 136.5, 130.5, 130.4, 129.8, 129.8, 128.5, 127.7, 127.6, 127.3, 127.3, 127.1, 126.3, 125.0, 117.5, 106.9, 106.8, 93.3, 93.2, 85.5, 85.1, 72.3, 72.0, 47.8, 47.5, 47.0, 46.8, 40.1, 39.8, 30.6, 30.3, 21.5, 20.9, 20.6, 9.6, 9.3;

HRMS-ESI: Exact mass calcd. for $C_{28}H_{33}O_3N_2S$ $[M+H]^+$: 477.2206; Found: 477.2205;

IR (KBr): 2942.90, 2359.04, 1653.84, 1595.22, 1452.17, 1344.10, 1224.03, 1201.37, 1156.61, 1091.72, 1024.58, 811.49, 764.92, 744.10, 704.67, 679.44, 656.88, 582.38.

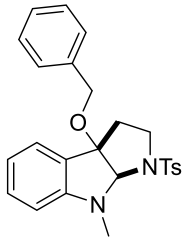

2s

2s Yellow solid, 29.4 mg, 34% yield, 14/1 dr;

For the mixture of the two diastereomers: **^{1}H NMR** (600 MHz, Chloroform-*d*) *δ* 7.76 (d, *J* = 8.0 Hz, 2 H), 7.73 (s, 0.07 H), 7.37 (s, 0.07 H), 7.33 - 7.22 (m, 6 + 0.56 H), 7.20 (d, *J* = 2.0 Hz, 0.07 H), 7.15 - 7.09 (m, 3 + 0.07 H),6.73 (t, *J* = 7.4 Hz, 1 H), 6.50 (d, *J* = 8.0 Hz, 0.07 H), 6.37 (d, *J* = 8.4 Hz, 1 H), 5.38 (s, 1 H), 5.36 (s, 0.07 H), 4.18

– 4.14 (m, 1 + 0.07 H), 4.06 - 4.04 (m, 1 + 0.07 H), 3.64 - 3.60 (m, 1 + 0.07 H), 3.16 - 3.11 (m, 1 + 0.07 H), 3.02 (s, 3 H), 2.99 (s, 0.21 H), 2.40 (s, 3 + 0.21 H), 2.14 - 2.10 (m, 1 + 0.07 H), 1.90 - 1.85 (m, 1 + 0.07 H);

^{13}C NMR (150 MHz, Chloroform-*d*) *δ* 151.9,143.7, 137.9, 136.2, 130.8, 129.8, 128.2, 127.62, 127.5, 127.3, 125.2, 124.0, 117.8, 108.1, 106.7, 93.8, 93.4, 86.3, 86.3, 67.6, 67.4, 47.5, 39.2, 31.2, 21.5;

HRMS-ESI: Exact mass calcd. for $C_{25}H_{26}O_3N_2NaS$ [M+Na$^+$]: 457.1556; Found: 457.1548;

IR(KBr): 2890.77, 2358.52, 1608.79, 1481.94, 1349.52, 1166.11, 1045.23, 1029.40, 1012.45, 983.52, 943.02, 844.94, 813.85, 755.47, 697.62, 661.00, 627.00, 594.40, 573.09, 546.75, 466.69.

5. Ligand and substrate preparation

5.1 Synthesis of the parent ligand L4

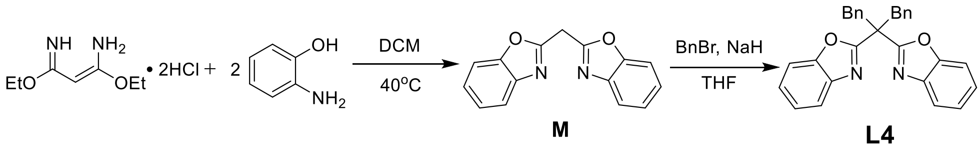

M

L4

2-Aminophenol (5.6 g, 50.0 mmol, 2.5 eq.) and ethylbisimidate dihydrochloride (4.62 g, 20 mmol, 1.0 eq.) were dissolved in DCM (50 mL). Then the reaction mixture was heated to 40°Cover night, after cooling to rt, stirred at -32 ℃ in a fridge. The resulting crystalline material was filtered off, washed subsequently with saturate aq. $NaHCO_3$ solution (2 × 50 mL). Then extracted with CH_2Cl_2 (3 × 20 mL), dried over anhydrous $NaSO_4$ and concentrated under reduced pressureto afford **M**.

The crude of **M** (1.0 g, 4.0 mmol, 1 eq.) was dissolved in THF (50 mL). NaH (0.64 g, 16.0 mmol, 4 eq.) was added in portions at 0 ℃ and stirred for 15 min. Then a solution of Benzyl bromide (2.74 g, 16.0 mmol, 4 eq.) was added dropwise at 0 °C. The reaction mixture was then allowed to worm up to room temperature and monitored by TLC. Upon completion, the reaction mixture was quenched with H_2O, extracted with ethyl acetate (3 × 20 mL), dried over anhydrous $NaSO_4$ and concentrated under reduced pressure. The crude material was purified by chromatography on silica gel (ethyl acetate /Petroleum ether 10/1 v/v) to afford **L4**.

^{1}H NMR (600 MHz, Chloroform-*d*) δ 7.80 (d, *J* = 12.0 Hz, 2 H), 7.46 (d, *J* = 6.0 Hz, 2 H), 7.40 -7.36 (m, 4 H), 7.16 (t, *J* = 6.0 Hz, 2 H), 7.10 (t, *J* = 6.0 Hz, 4 H), 6.88 (d, *J* = 12.0 Hz, 4 H), 3.91 (s, 4 H);

^{13}C NMR (150 MHz, Chloroform-*d*) δ 165.5, 150.6, 140.7, 135.5, 130.0, 128.2, 127.0, 125.3, 124.5, 120.3, 110.8, 50.5, 41.7;

HRMS-ESI: Exact mass calcd. for $C_{29}H_{23}O_2N_2$ $[M+H]^+$: 431.1754; Found: 431.1749;

IR (KBr): 2919.70, 1354.79, 1166.75, 1083.65, 1058.73, 995.09, 912.07, 865.88, 726.73, 659.13, 597.82, 549.61.

5.2 Preparation of cyclopropyl substituted tryptamines

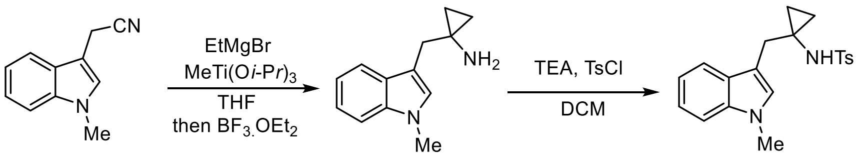

1n

To a solution of nitrile (508.0 mg, 2.95 mmol) and MeTi(O*i*-Pr)$_3$ (1.33 mL, 1.5 eq.) in THF (30 mL) was dropwise added under argon EtMgBr (ca 2M in Et_2O, 1.5 eq.). The yellow solution darkened gradually within 5 minutes. After stirring for 1.5 h, BF_3•OEt_2 was added (750 μL, 2.0 eq.). After 30 min, the dark mixture was quenched with HCl 1 M (10 mL). EtOAc (20 mL) was added, followed by NaOH 3M (10 mL). The mixture was stirred until the blue aqueous solution becomes white. The aqueous phase was extracted twice with ethyl acetate. After drying by $NaSO_4$ and evaporation of the solvents, the crude material was purified by flash-chromatography ethyl acetate.

^{1}H NMR (600 MHz, Chloroform-*d*) δ 7.76 (d, J = 6.0 Hz, 2 H), 7.46 (d, J = 6.0 Hz, 1 H), 7.33-7.29 (m, 1 H), 7.25 (t, J = 6.0 Hz, 1 H), 7.12 (t, J = 6.0 Hz, 1 H), 6.87 (s, 1 H), 3.78 (s, 3 H), 2.86 (s, 2 H), 2.46 (s, 3 H), 0.90 (t, J = 6.0 Hz, 2 H), 0.75 (t, J = 6.0 Hz, 2 H);

^{13}C NMR (150 MHz, Chloroform-*d*) δ 143.3, 139.3, 137.0, 129.6, 128.0, 127.2, 121.6, 119.3, 118.9, 110.2, 109.2, 35.8, 32.8, 32.7, 21.5, 13.0;

HRMS-ESI: Exact mass calcd. for $C_{20}H_{23}O_2N_2S$ $[M+H]^+$: 355.1475; Found: 355.1466;

IR (KBr): 3137.56, 1654.50, 1401.77, 1151.60, 813.44, 731.90, 664.52, 555.15.

6. Synthesis of CPC-1and Product Characterizations

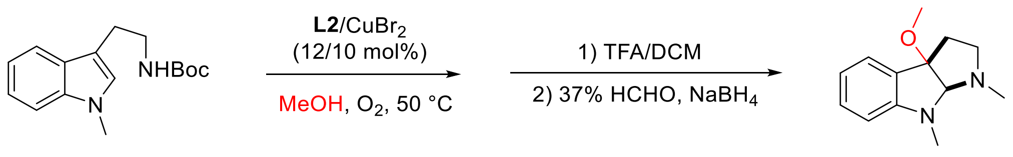

CPC-1

A mixture of $CuBr_2$ (4.46 mg, 0.02 mmol), **L2** (6.68 mg, 0.024 mmol), and **1s** (54.9 mg, 0.2 mmol) were added in a dried Schlenk tube before oxygen replacement operation, then MeOH (2 ml) was added under O_2 atmosphere, and the reaction system was stirred at 50 ℃ for 36 h. After completion as detected by TLC, the reaction was filtered through a glass funnel with thin layer (30 mm) of silica gel (300-400 mesh) and eluted with DCM/Ethyl Acetate. The filtrate was concentrated under reduced pressure, purified by flash chromatography (Petroleum ether/ethyl acetate = 10/1) to afford cycloadduct intermediate as white solid (44.4 mg, 73% yield).

Chargecycloadduct intermediate (60.1mg, 0.2 mmol) dissolved in anhydrousCH_2Cl_2 (2.5 mL) after raw material accumulation, TFA (2.5 mL) was added before stirred at room temperature for 4.5 hours. The mixture was concentrated under reduced pressure without further purified. The crude residue was dissolved in anhydrous MeOH after dried，and Formalin (26.0 mg，0.32 mmol) were added under argon atmosphere, then sodium borohydride (22.7 mg, 0.6mmol) were added before reaction mixture was stirred for 10 minutes at room temperature. After 30 min, the reaction mixture was quenched with H_2O (50 mL), extracted with EtOAc. The organic layers were combined and dried over Na_2SO_4, filtered, concentrated in vacuum. The residue was purified by chromatography on silica gel (CH_2Cl_2/MeOH, 20:1) to afford (±)-CPC-1 as an oil (32.2 mg, 74% yield).

(±)-CPC-1: ^{1}**H NMR** (600 MHz, Chloroform-*d*) *δ* 7.27 - 7.26 (m, 0.26 H), 7.23 (d, *J* = 2.4 Hz, 0.13 H),7.21 - 7.18 (m, 1 H), 7.15 (d, *J* = 6.0 Hz, 1 H), 6.74 (t, *J* = 6.0 Hz, 1 H), 6.50 (d, *J* = 6.0 Hz, 1 H), 6.35 (d, *J* = 12.0 Hz, 0.13 H), 4.36 (s, 1 H), 3.05 (s, 0.39 H), 3.04 (s, 3 H), 2.94 (s, 0.39 H), 2.97 (s, 3 H), 2.81 - 2.78 (m, 1 + 0.13 H), 2.64 -2.61 (m, 1 + 0.13 H), 2.57 - 2.56 (m, 3 + 0.39 H), 2.37 - 2.33 (m, 1 + 0.13 H), 2.15 -

2.11 (m, 1 + 0.13 H).

^{13}C NMR (150 MHz, Chloroform-*d*) δ 153.1, 129.7, 128.0, 124.1, 117.9, 107.8, 94.0, 91.7, 52.5, 39.3, 38.6, 36.3. All spectral data were in agreement with the literature (*Org.Biomol. Chem.* 2020, **18**, 32-35).

7. ^{1}H NMR and ^{13}C NMR Spectra of compounds

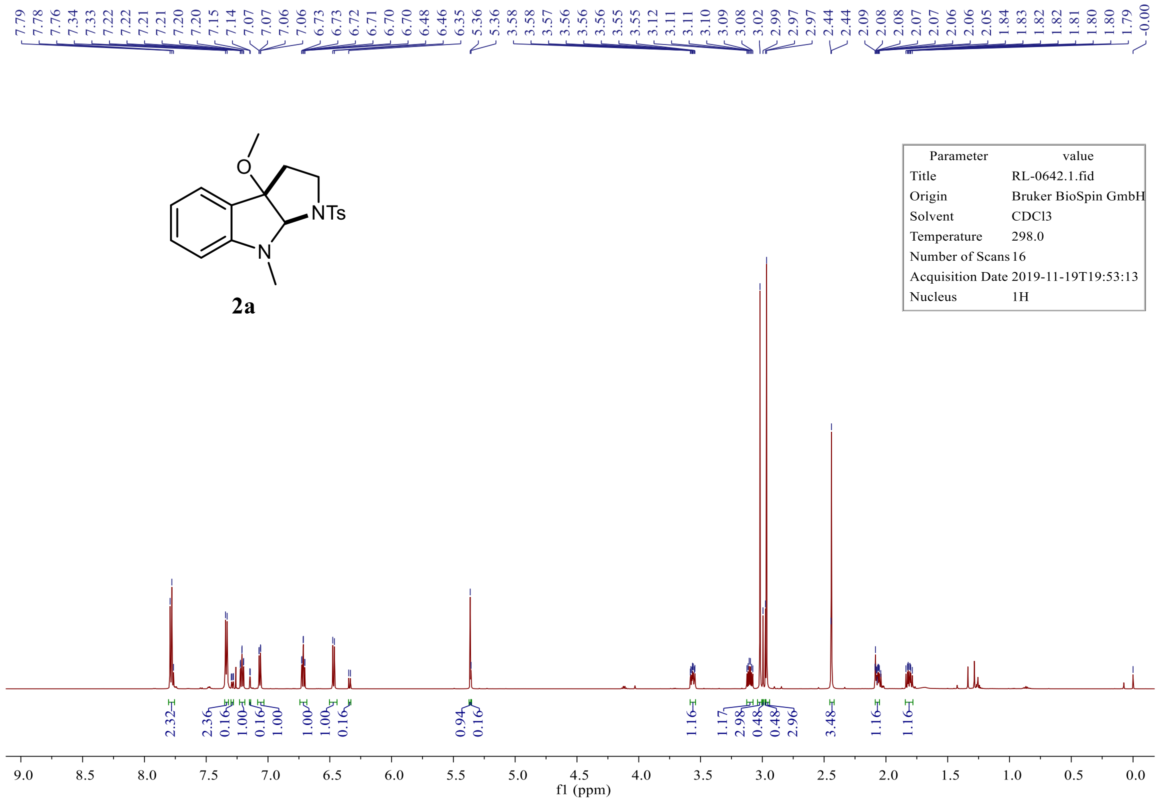

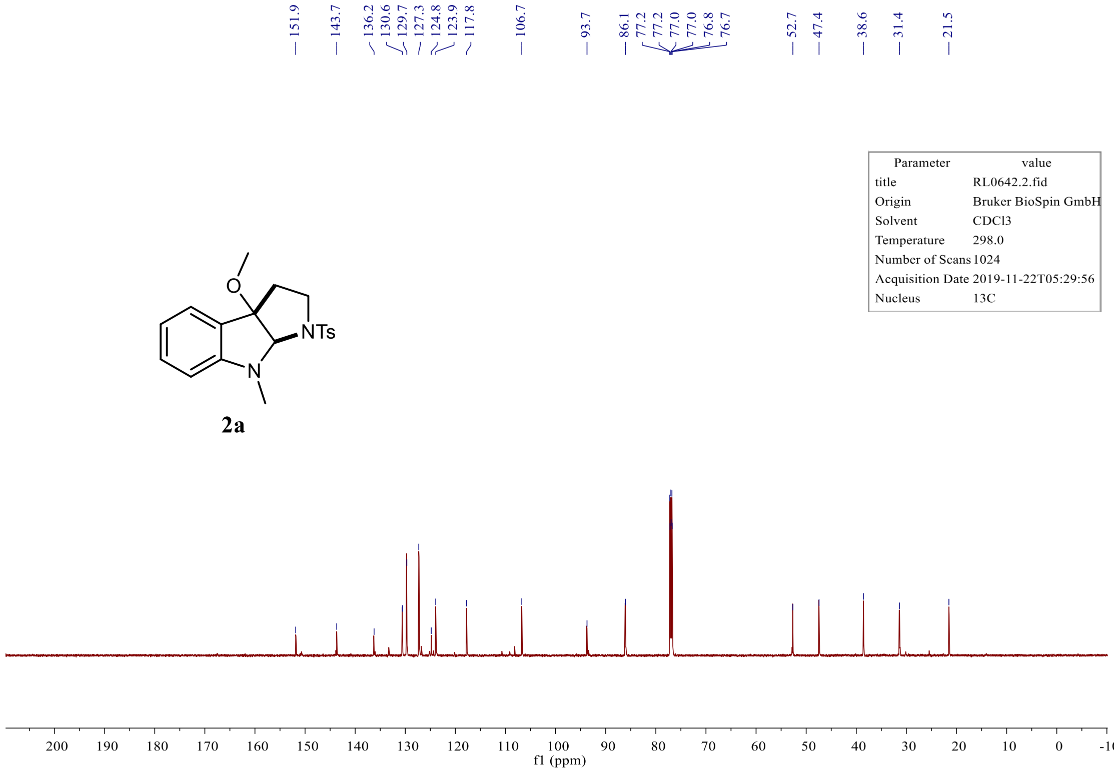

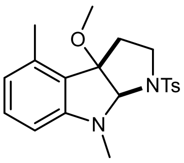

2b

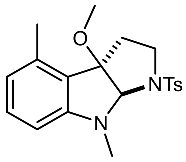

2b'

Parameter	value
title	RL0788.1.fid
Origin	Bruker BioSpin GmbH
Solvent	CDCl3
Temperature	300.6
Number of Scans	4
Acquisition Date	2020-05-18T14:45:21
Nucleus	1H

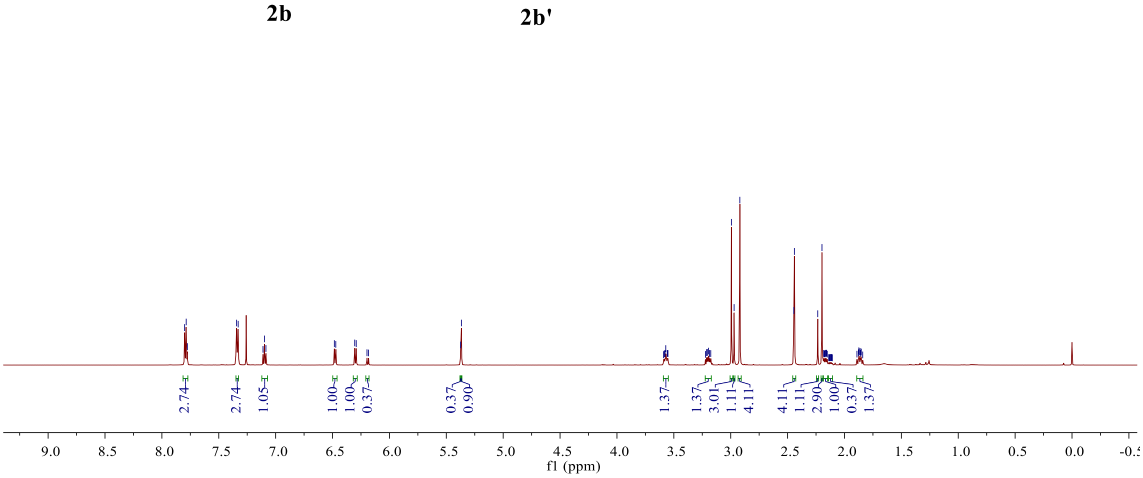

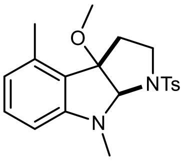

2b

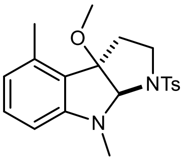

2b'

Parameter	value
title	RL0788.2.fid
Origin	Bruker BioSpin GmbH
Solvent	CDCl3
Temperature	300.7
Number of Scans	32
Acquisition Date	2020-05-18T14:47:37
Nucleus	13C

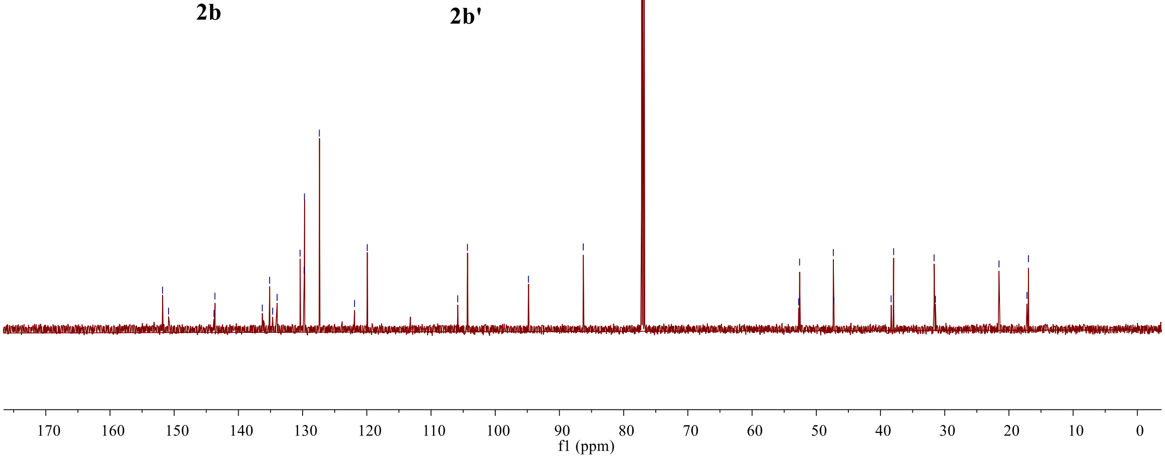

Parameter	value
title	RL0784.1.fid
Origin	Bruker BioSpin GmbH
Solvent	CDCl3
Temperature	298.0
Number of Scans	16
Acquisition Date	2020-05-12T16:12:08
Nucleus	1H

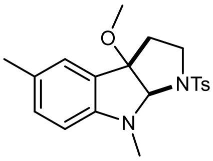

2c

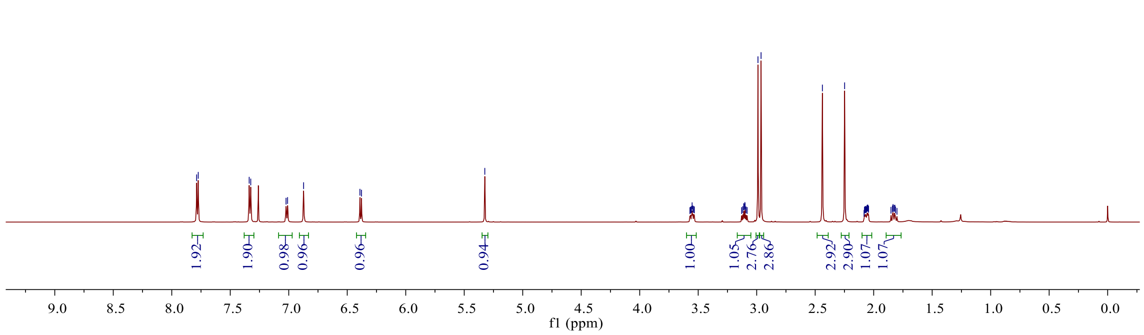

Parameter	value
title	RL0784.2.fid
Origin	Bruker BioSpin GmbH
Solvent	CDCl3
Temperature	298.1
Number of Scans	32
Acquisition Date	2020-05-12T16:14:20
Nucleus	13C

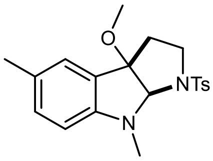

2c

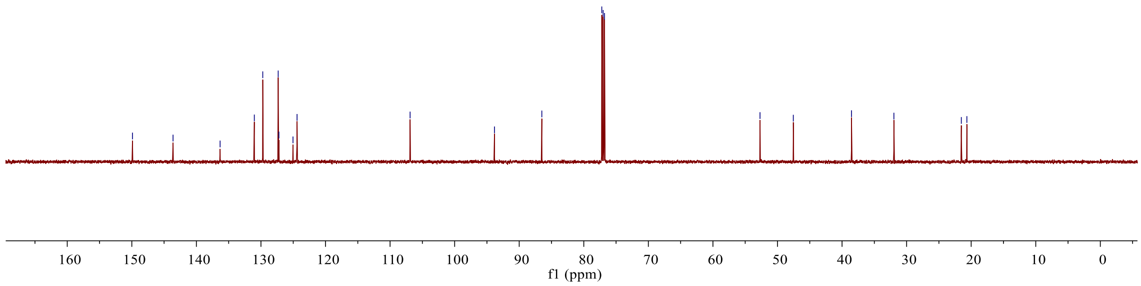

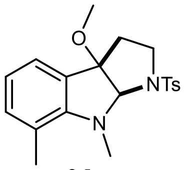

2d

Parameter	value
title	RL0781.1.fid
Origin	Bruker BioSpin GmbH
Solvent	CDCl3
Temperature	298.0
Number of Scans	16
Acquisition Date	2020-05-12T16:05:11
Nucleus	1H

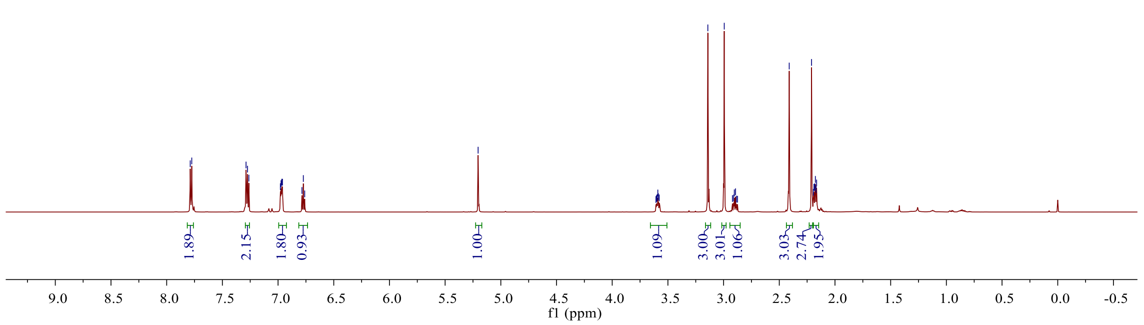

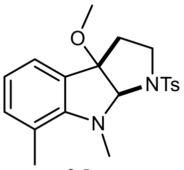

2d

Parameter	value
title	RL0781.2.fid
Origin	Bruker BioSpin GmbH
Solvent	CDCl3
Temperature	298.1
Number of Scans	32
Acquisition Date	2020-05-12T16:07:23
Nucleus	13C

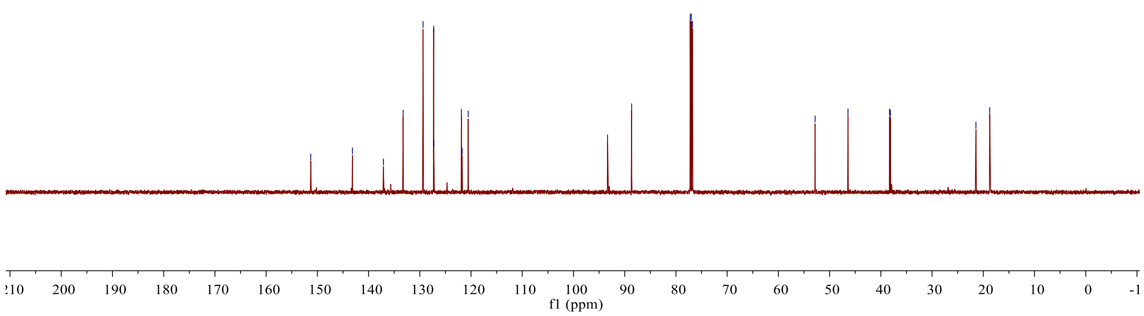

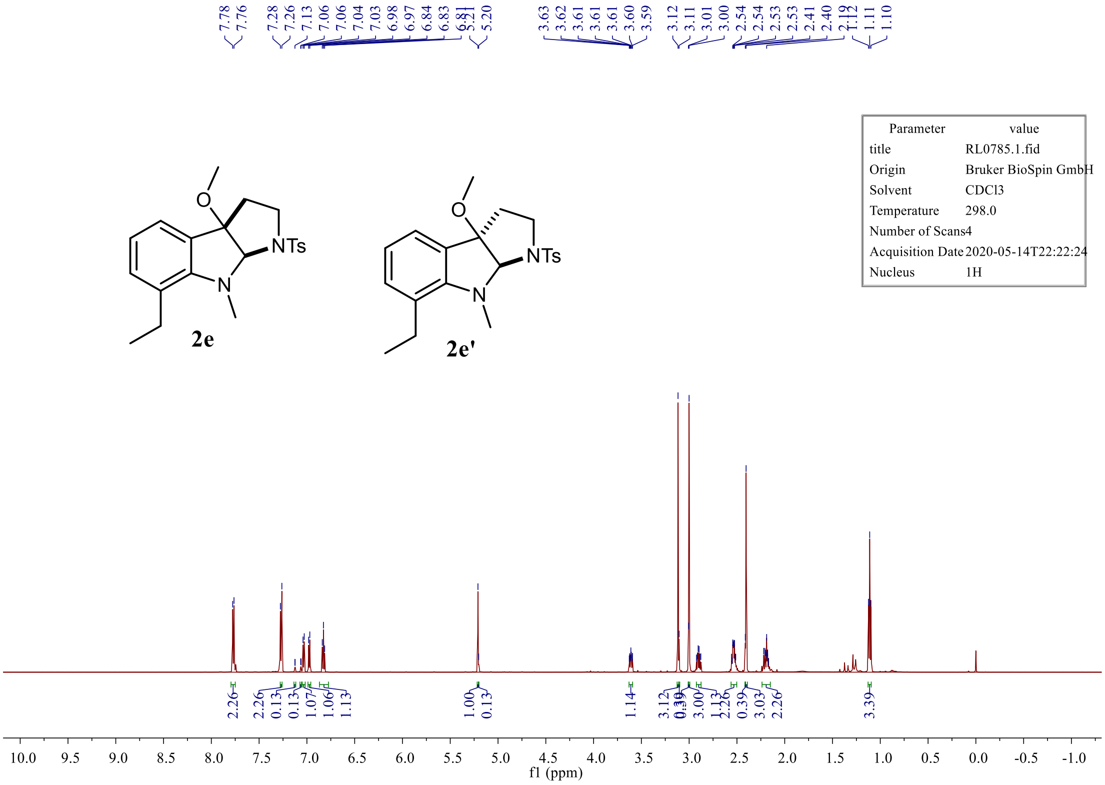
7.78
7.76
7.28
7.26
7.13
7.06
7.06
7.04
7.03
6.98
6.97
6.84
6.83
6.81
5.21
5.20
3.63
3.62
3.61
3.61
3.61
3.60
3.59
3.12
3.11
3.01
3.00
2.54
2.54
2.53
2.53
2.41
2.40
2.19
1.12
1.11
1.10
NTs
N
2e
2e'
Parameter value
title RL0785.1.fid
Origin Bruker BioSpin GmbH
Solvent CDCl3
Temperature 298.0
Number of Scans 4
Acquisition Date 2020-05-14T22:22:24
Nucleus 1H
2.26
2.26
0.13
0.13
1.07
1.06
1.13
1.00
0.13
1.14
3.12
0.39
3.00
1.13
2.26
0.39
3.03
2.26
3.39
f1 (ppm)

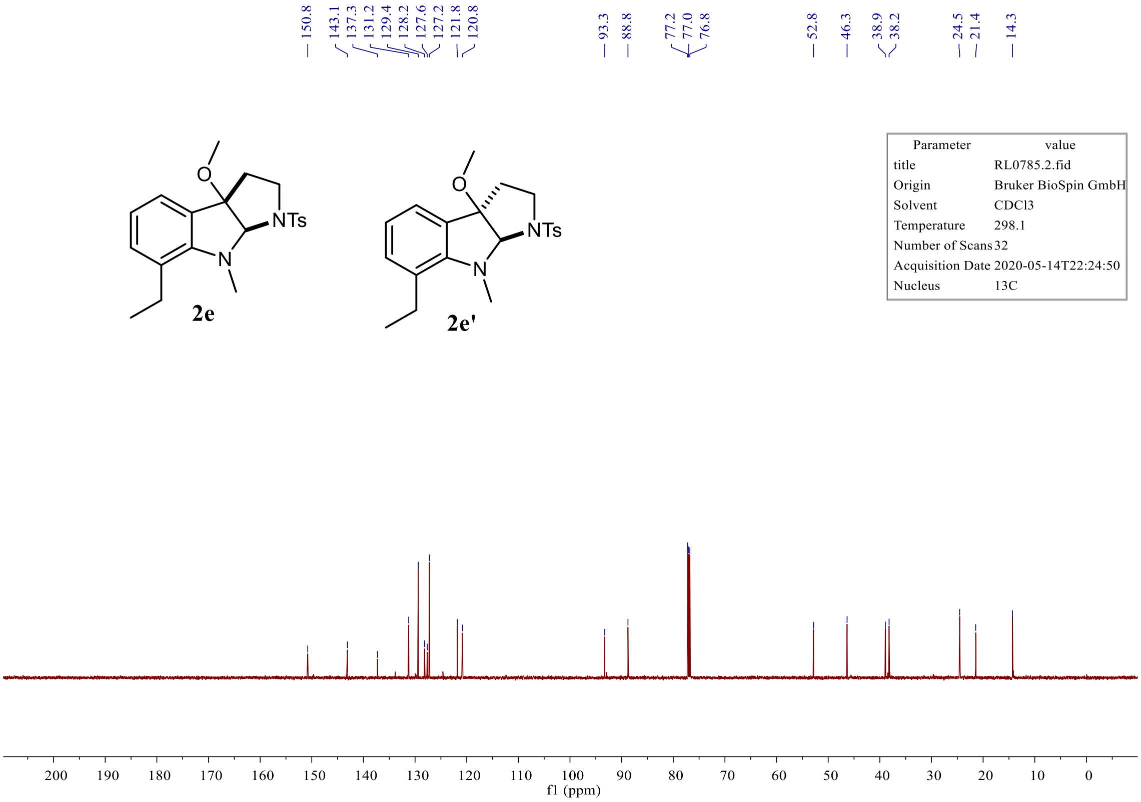
150.8
143.1
137.3
131.2
129.4
128.2
127.6
127.2
121.8
120.8
93.3
88.8
77.2
77.0
76.8
52.8
46.3
38.9
38.2
24.5
21.4
14.3
NTs
N
2e
2e'
Parameter value
title RL0785.2.fid
Origin Bruker BioSpin GmbH
Solvent CDCl3
Temperature 298.1
Number of Scans 32
Acquisition Date 2020-05-14T22:24:50
Nucleus 13C
f1 (ppm)

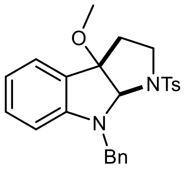

2f

Parameter	value
title	RL-0720.1.fid
Origin	Bruker BioSpin GmbH
Solvent	CDCl3
Temperature	298.0
Number of Scans	16
Acquisition Date	2020-01-13T20:02:54
Nucleus	1H

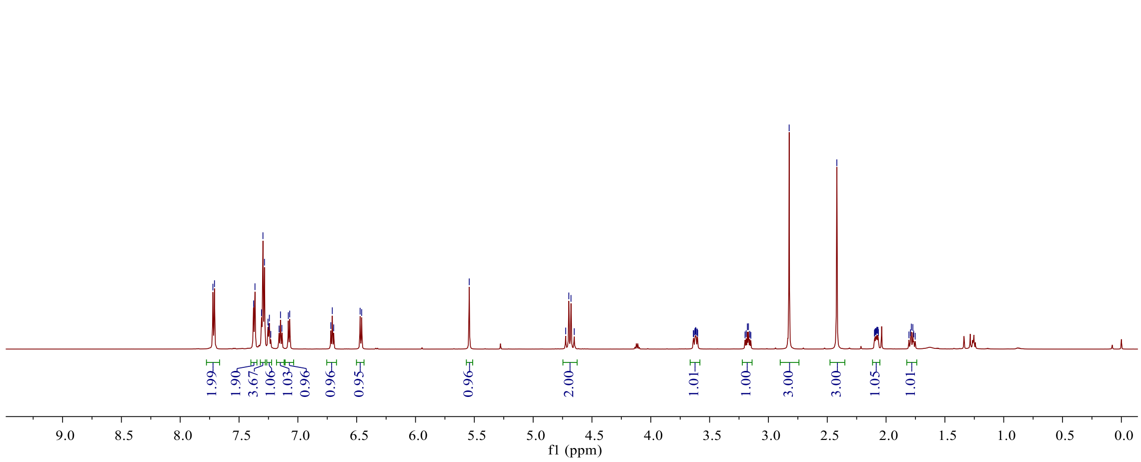

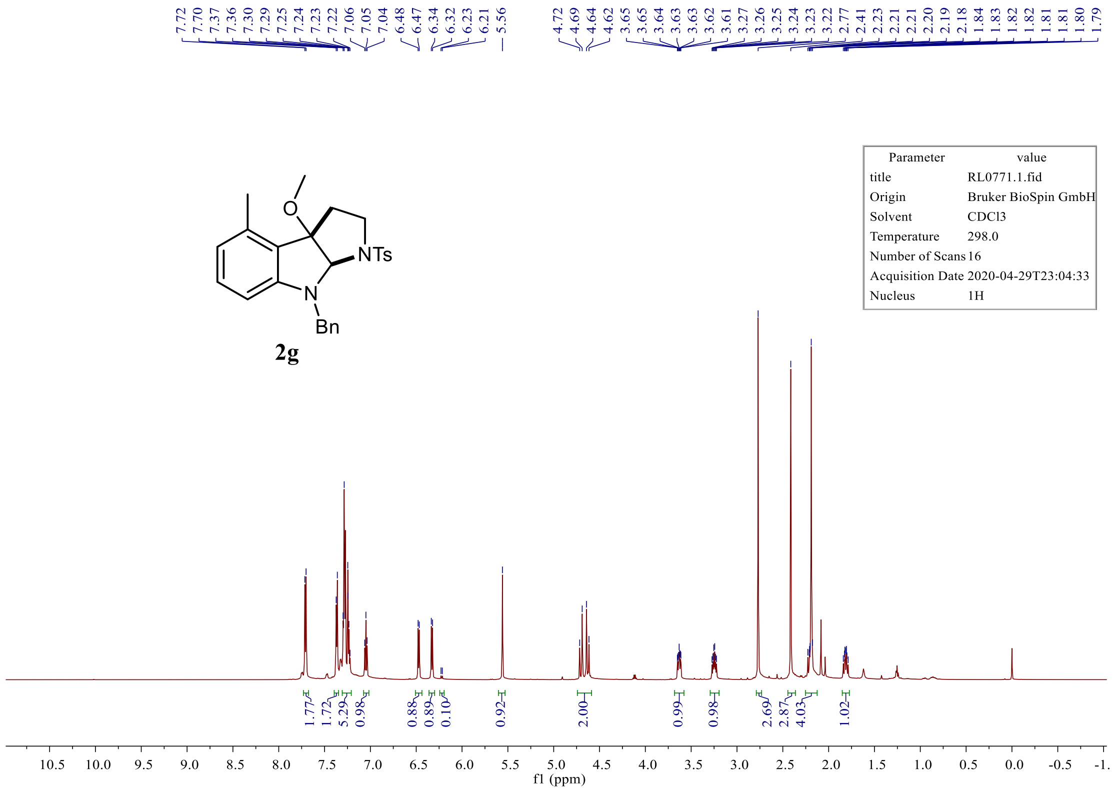
NTs
N
Bn
2g
Parameter value
title RL0771.1.fid
Origin Bruker BioSpin GmbH
Solvent CDCl3
Temperature 298.0
Number of Scans 16
Acquisition Date 2020-04-29T23:04:33
Nucleus 1H
f1 (ppm)

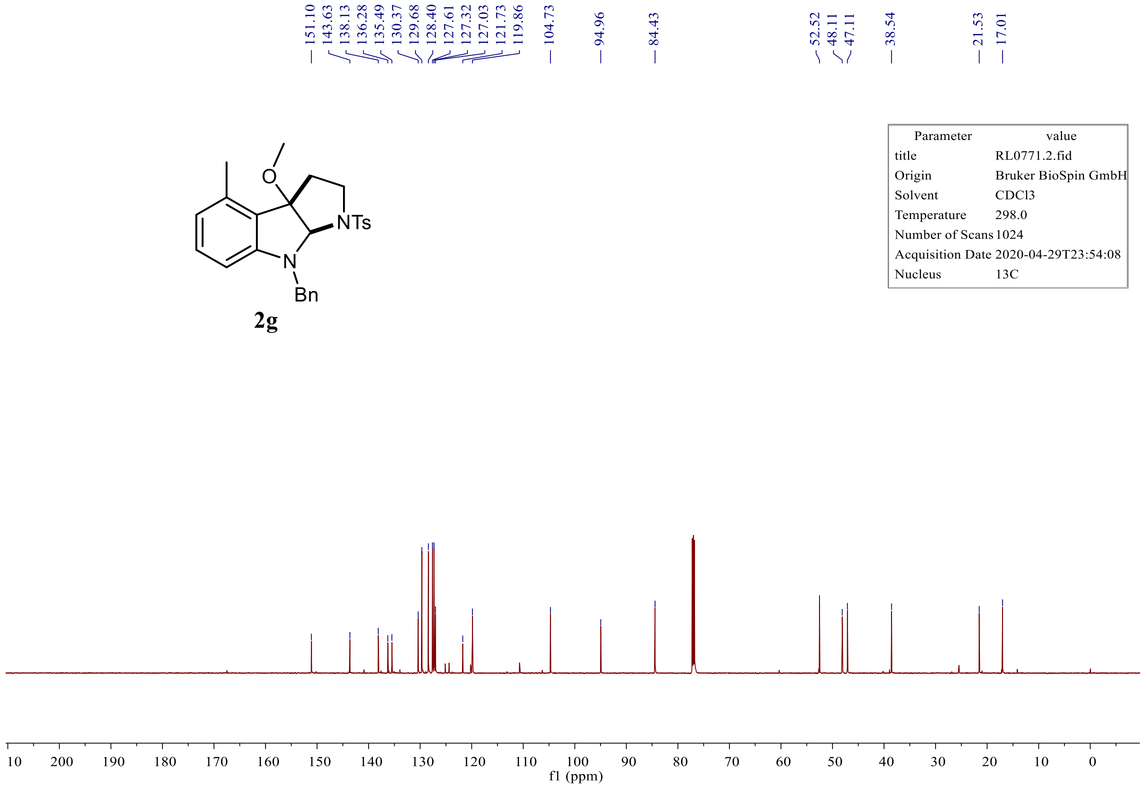
NTs
N
Bn
2g
Parameter value
title RL0771.2.fid
Origin Bruker BioSpin GmbH
Solvent CDCl3
Temperature 298.0
Number of Scans 1024
Acquisition Date 2020-04-29T23:54:08
Nucleus 13C
f1 (ppm)

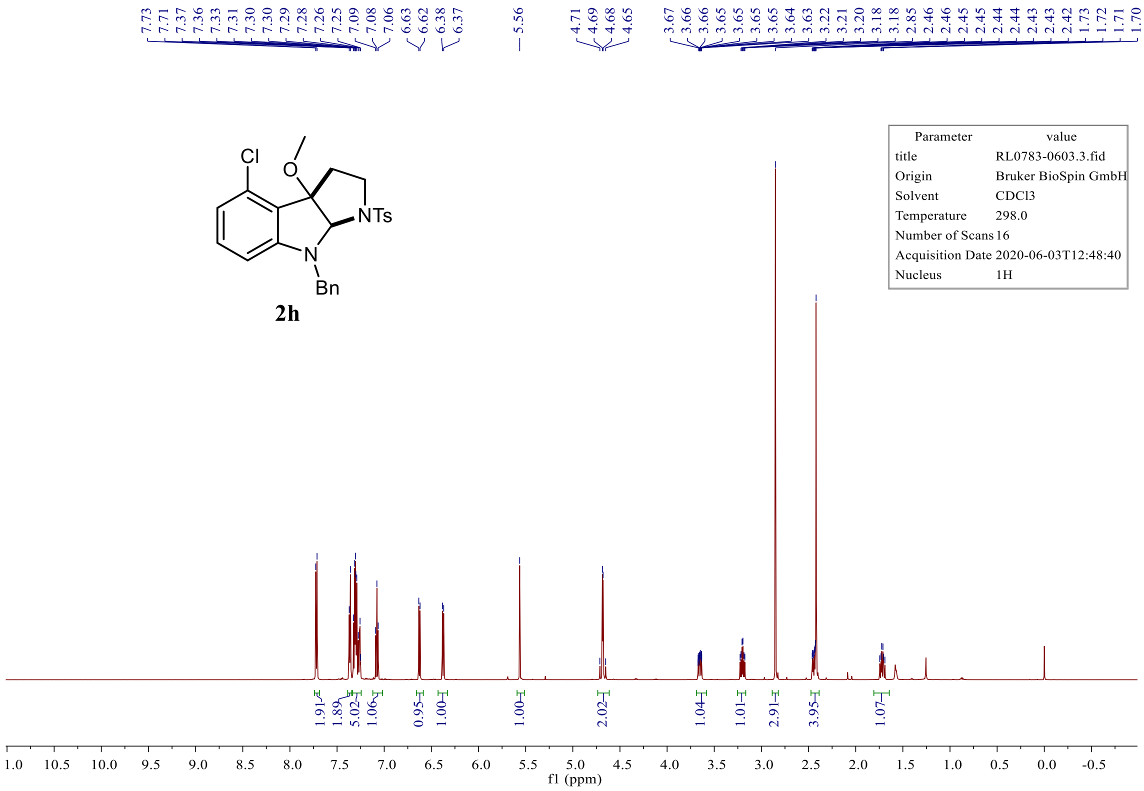
7.73 7.71 7.37 7.36 7.33 7.31 7.30 7.30 7.29 7.28 7.26 7.25 7.09 7.08 7.06 6.63 6.62 6.38 6.37 5.56 4.71 4.69 4.68 4.65 3.67 3.66 3.66 3.65 3.65 3.65 3.65 3.64 3.63 3.22 3.21 3.20 3.18 3.18 2.85 2.46 2.46 2.45 2.45 2.44 2.44 2.43 2.43 2.42 1.73 1.72 1.71 1.70
Cl
O
NTs
N
Bn
2h
Parameter value
title RL0783-0603.3.fid
Origin Bruker BioSpin GmbH
Solvent CDCl3
Temperature 298.0
Number of Scans 16
Acquisition Date 2020-06-03T12:48:40
Nucleus 1H
1.91 1.89 5.02 1.06 0.95 1.00 1.00 2.02 1.04 1.01 2.91 3.95 1.07
f1 (ppm)

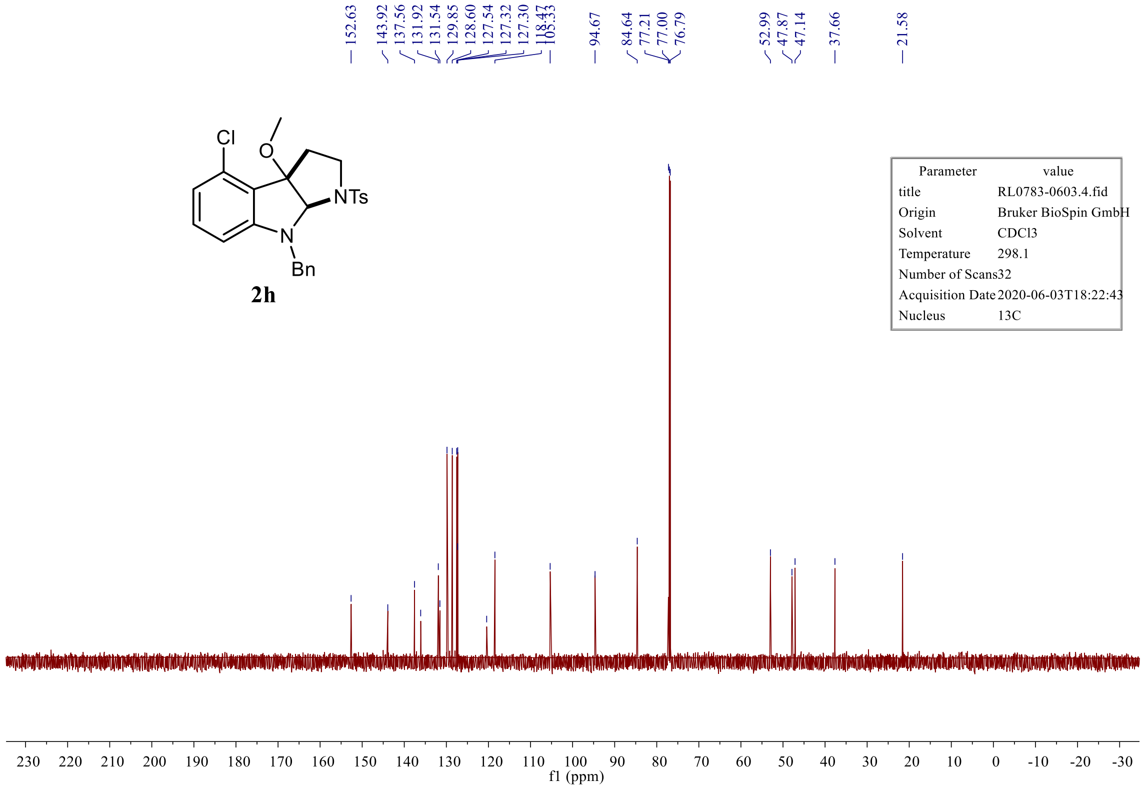
152.63 143.92 137.56 131.92 131.54 129.85 128.60 127.54 127.32 127.30 118.47 105.33 94.67 84.64 77.21 77.00 76.79 52.99 47.87 47.14 37.66 21.58
Cl
O
NTs
N
Bn
2h
Parameter value
title RL0783-0603.4.fid
Origin Bruker BioSpin GmbH
Solvent CDCl3
Temperature 298.1
Number of Scans 32
Acquisition Date 2020-06-03T18:22:43
Nucleus 13C
f1 (ppm)

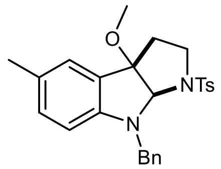

2i

Parameter	value
title	rl0773.1.fid
Origin	Bruker BioSpin GmbH
Solvent	CDCl3
Temperature	298.0
Number of Scans	16
Acquisition Date	2020-05-07T15:26:08
Nucleus	1H

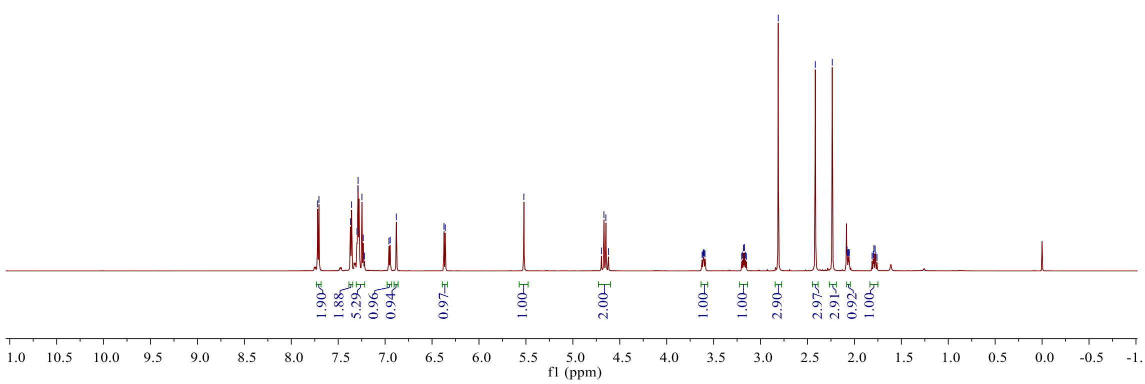

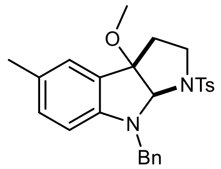

2i

Parameter	value
title	RL-0773-20200509.2.fid
Origin	Bruker BioSpin GmbH
Solvent	CDCl3
Temperature	298.0
Number of Scans	100
Acquisition Date	2020-05-08T18:05:12
Nucleus	13C

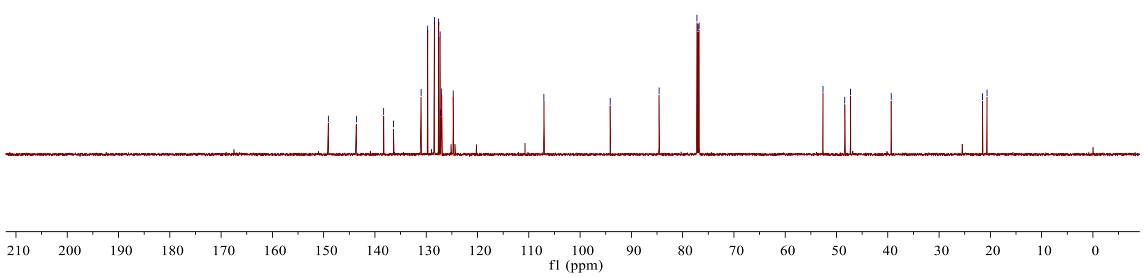

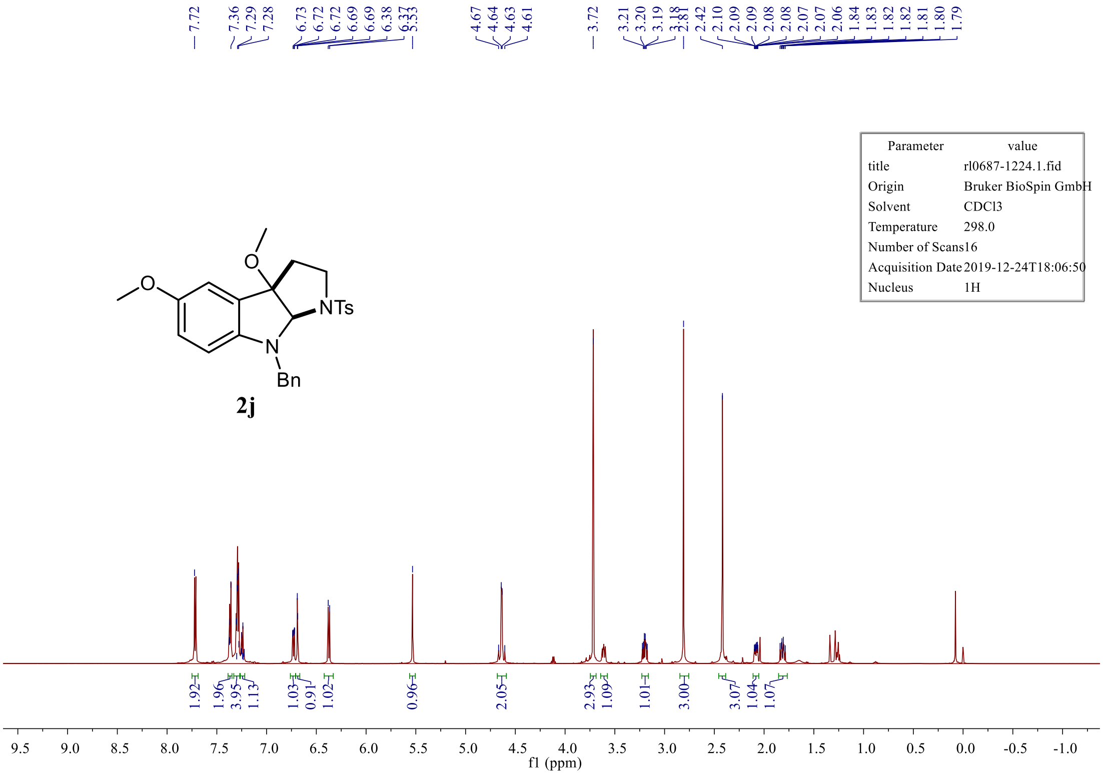
O
O
NTs
N
Bn
2j
Parameter value
title rl0687-1224.1.fid
Origin Bruker BioSpin GmbH
Solvent CDCl3
Temperature 298.0
Number of Scans16
Acquisition Date 2019-12-24T18:06:50
Nucleus 1H
f1 (ppm)

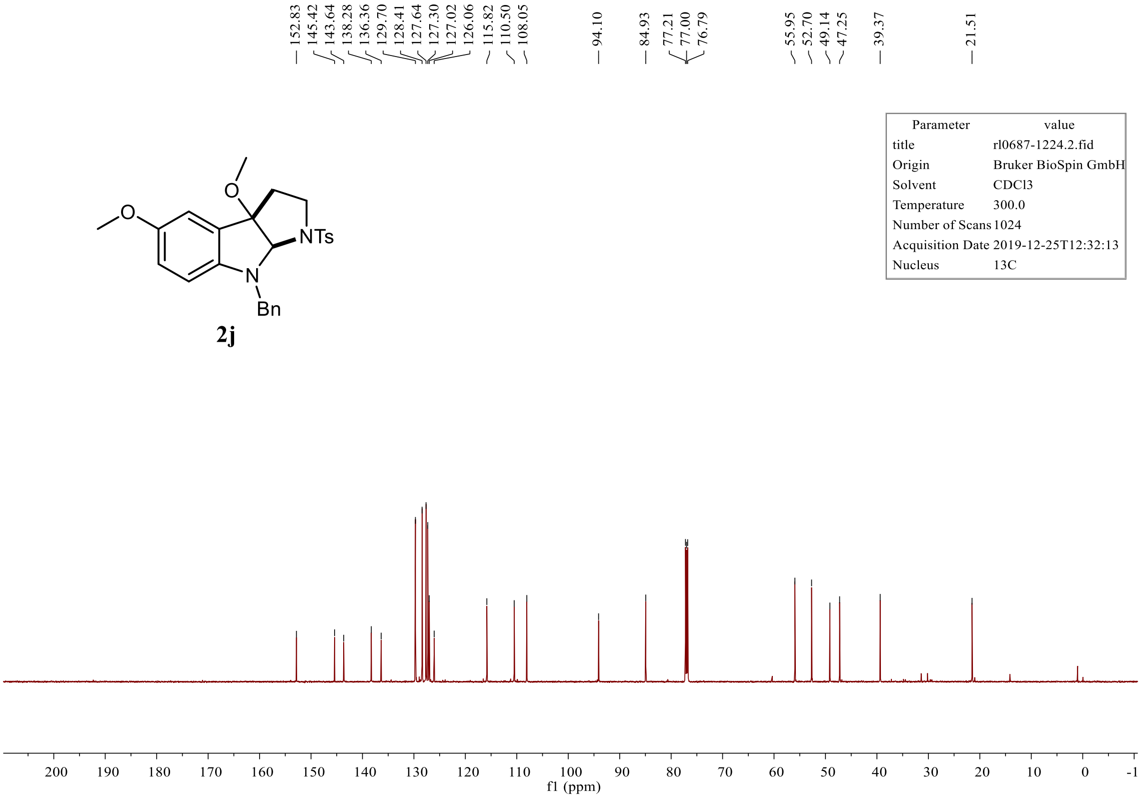
O
O
NTs
N
Bn
2j
Parameter value
title rl0687-1224.2.fid
Origin Bruker BioSpin GmbH
Solvent CDCl3
Temperature 300.0
Number of Scans 1024
Acquisition Date 2019-12-25T12:32:13
Nucleus 13C
f1 (ppm)

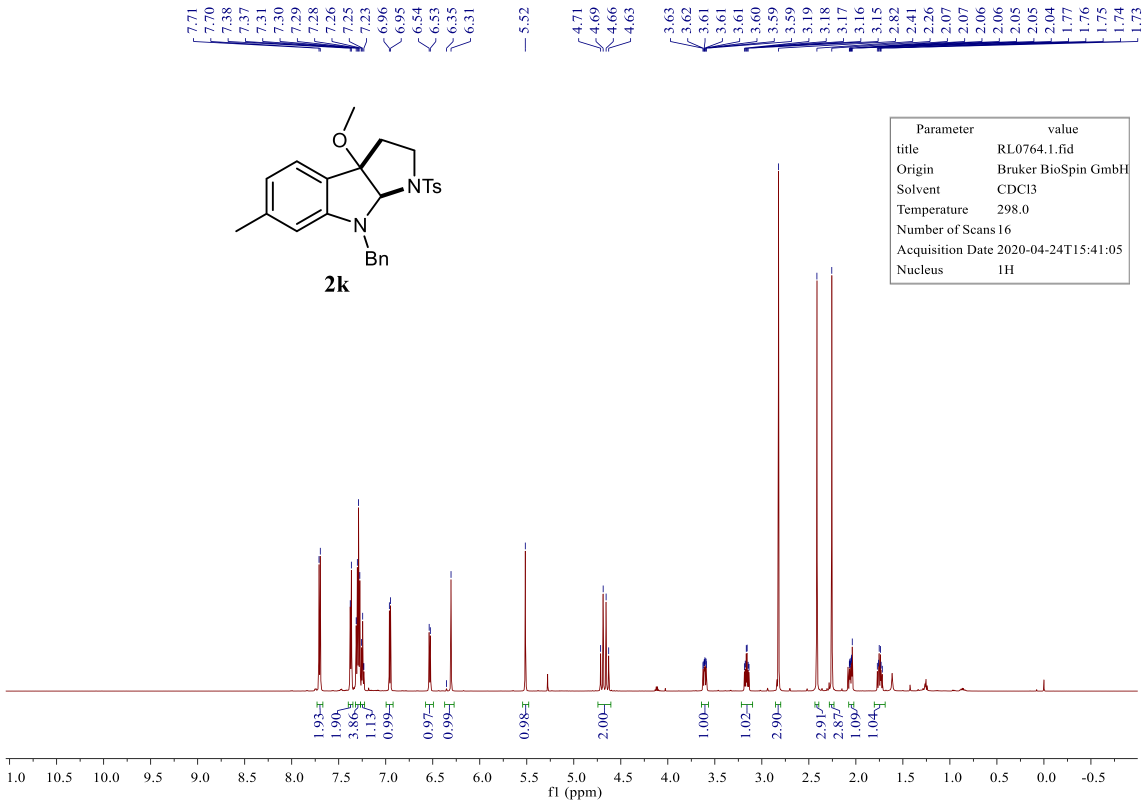
7.71 7.70 7.38 7.37 7.31 7.30 7.29 7.28 7.26 7.25 7.23 6.96 6.95 6.54 6.53 6.35 6.31 5.52 4.71 4.69 4.66 4.63 3.63 3.62 3.61 3.61 3.61 3.60 3.59 3.59 3.19 3.18 3.17 3.16 3.15 2.82 2.41 2.26 2.07 2.07 2.06 2.06 2.05 2.05 2.04 1.77 1.76 1.75 1.74 1.73
O
NTs
N
Bn
2k
Parameter value
title RL0764.1.fid
Origin Bruker BioSpin GmbH
Solvent CDCl3
Temperature 298.0
Number of Scans 16
Acquisition Date 2020-04-24T15:41:05
Nucleus 1H
1.93 1.90 3.86 1.13 0.99 0.97 0.99 0.98 2.00 1.00 1.02 2.90 2.91 2.87 1.09 1.04
1.0 10.5 10.0 9.5 9.0 8.5 8.0 7.5 7.0 6.5 6.0 5.5 5.0 4.5 4.0 3.5 3.0 2.5 2.0 1.5 1.0 0.5 0.0 -0.5
f1 (ppm)

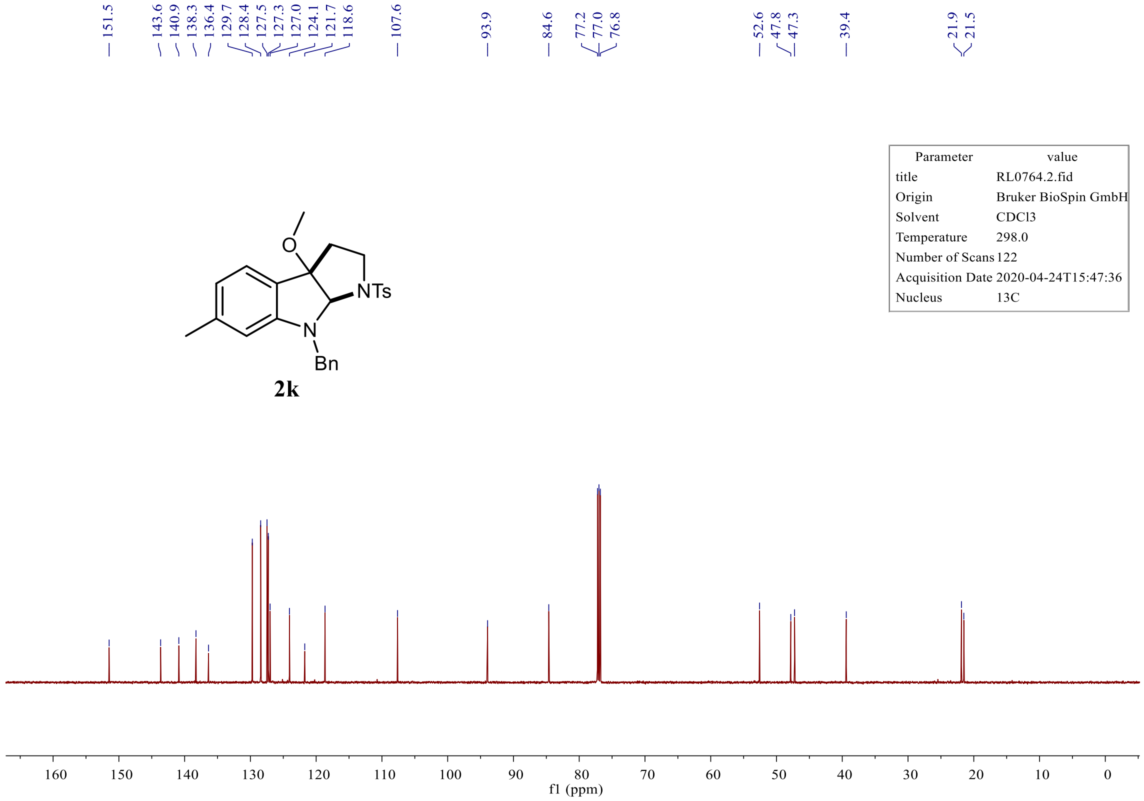
151.5 143.6 140.9 138.3 136.4 129.7 128.4 127.5 127.3 127.0 124.1 121.7 118.6 107.6 93.9 84.6 77.2 77.0 76.8 52.6 47.8 47.3 39.4 21.9 21.5
Parameter value
title RL0764.2.fid
Origin Bruker BioSpin GmbH
Solvent CDCl3
Temperature 298.0
Number of Scans 122
Acquisition Date 2020-04-24T15:47:36
Nucleus 13C
O
NTs
N
Bn
2k
160 150 140 130 120 110 100 90 80 70 60 50 40 30 20 10 0
f1 (ppm)

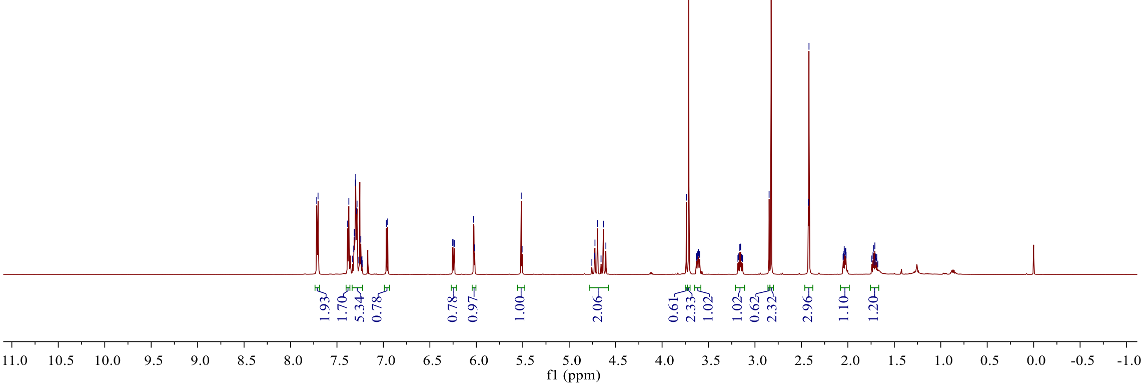

Parameter	value
title	RL0786.1.fid
Origin	Bruker BioSpin GmbH
Solvent	CDCl3
Temperature	298.0
Number of Scans	16
Acquisition Date	2020-05-15T17:42:15
Nucleus	1H

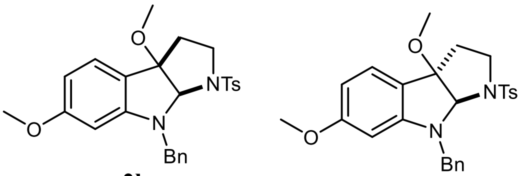

2l **2l′**

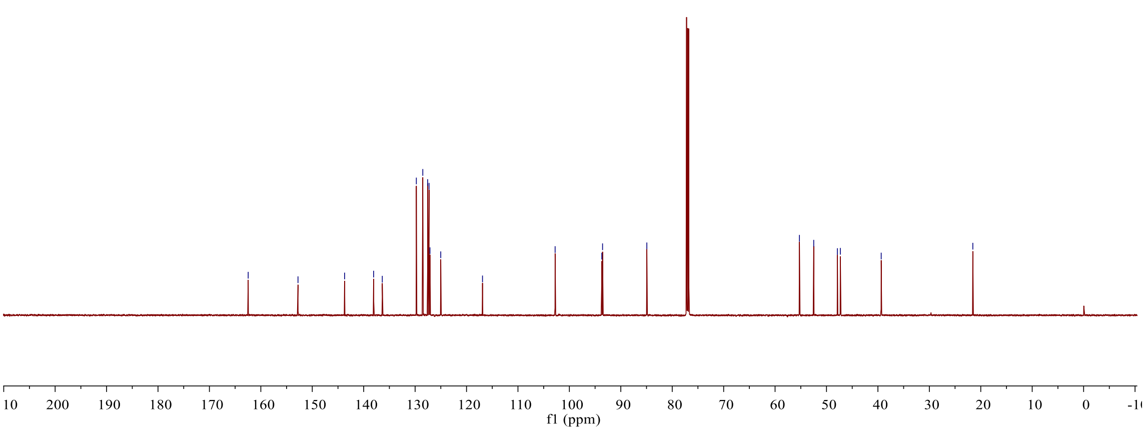

Parameter	value
title	RL0786.1.fid
Origin	Bruker BioSpin GmbH
Solvent	CDCl3
Temperature	298.0
Number of Scans	1024
Acquisition Date	2020-06-18T03:45:46
Nucleus	13C

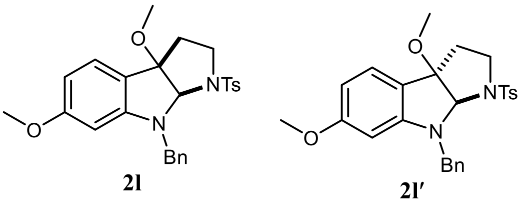

2l **2l′**

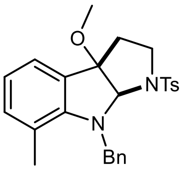

2m

Parameter	value
title	RL0761.2.fid
Origin	Bruker BioSpin GmbH
Solvent	CDCl3
Temperature	299.0
Number of Scans	16
Acquisition Date	2020-04-21T12:16:14
Nucleus	1H

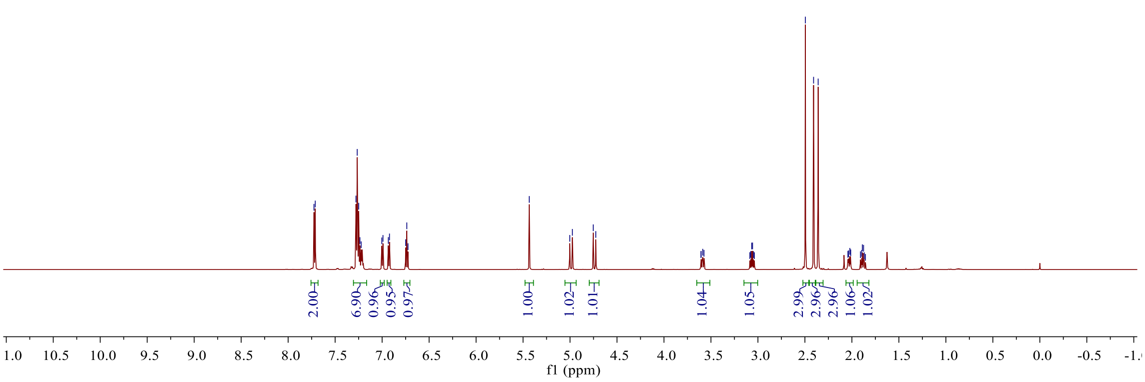

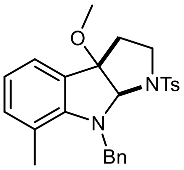

2m

Parameter	value
title	RL0761.3.fid
Origin	Bruker BioSpin GmbH
Solvent	CDCl3
Temperature	299.0
Number of Scans	758
Acquisition Date	2020-04-22T10:30:57
Nucleus	13C

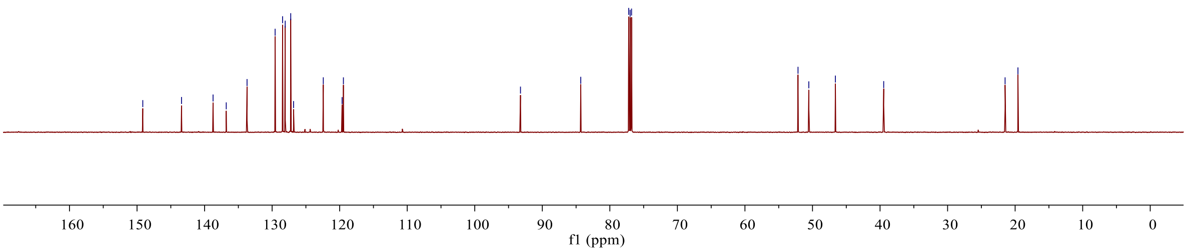

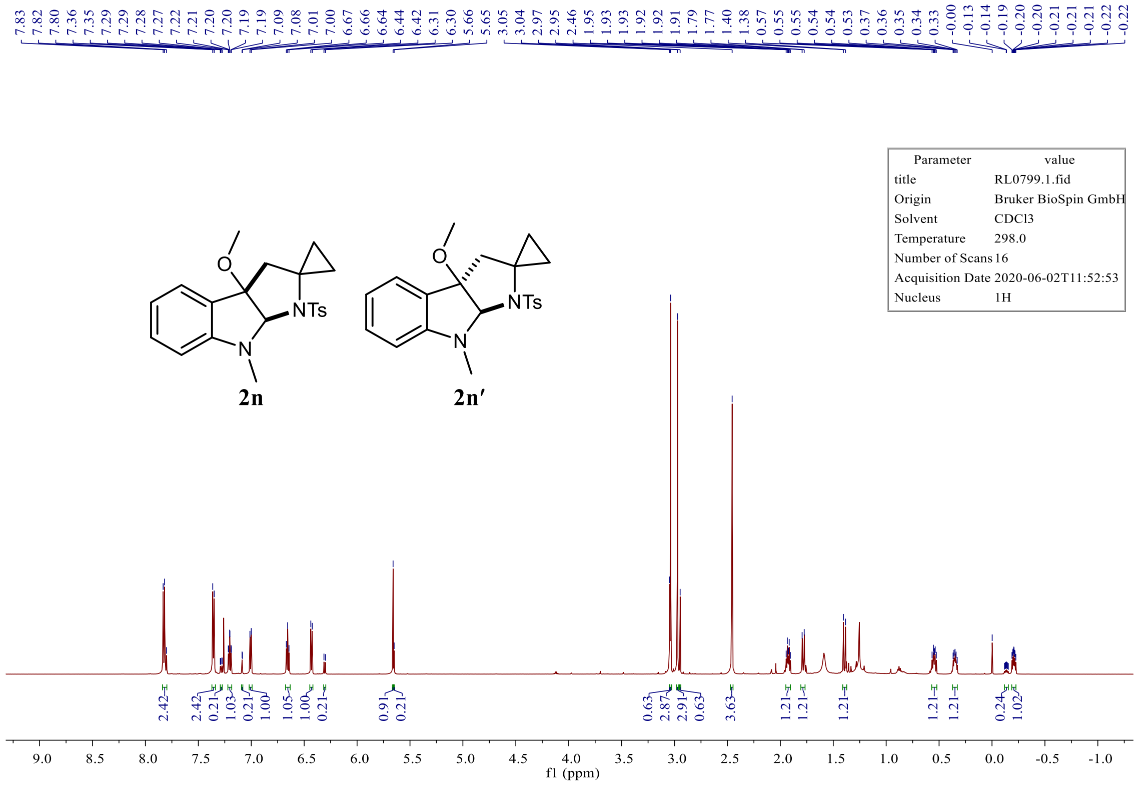
7.83 7.82 7.80 7.36 7.35 7.29 7.29 7.28 7.27 7.22 7.21 7.20 7.20 7.19 7.19 7.09 7.08 7.01 7.00 6.67 6.66 6.64 6.44 6.42 6.31 6.30 5.66 5.65 3.05 3.04 2.97 2.95 2.46 1.95 1.93 1.93 1.92 1.92 1.91 1.79 1.77 1.40 1.38 0.57 0.55 0.55 0.54 0.54 0.53 0.37 0.36 0.35 0.34 0.33 -0.00 -0.13 -0.14 -0.19 -0.20 -0.20 -0.21 -0.21 -0.21 -0.22 -0.22
Parameter value
title RL0799.1.fid
Origin Bruker BioSpin GmbH
Solvent CDCl3
Temperature 298.0
Number of Scans 16
Acquisition Date 2020-06-02T11:52:53
Nucleus 1H
O
NTs
N
2n
2n′
2.42 2.42 0.21 1.03 0.21 1.00 1.05 1.00 0.21 0.91 0.21 0.63 2.87 2.91 0.63 3.63 1.21 1.21 1.21 1.21 1.21 0.24 1.02
9.0 8.5 8.0 7.5 7.0 6.5 6.0 5.5 5.0 4.5 4.0 3.5 3.0 2.5 2.0 1.5 1.0 0.5 0.0 -0.5 -1.0
f1 (ppm)

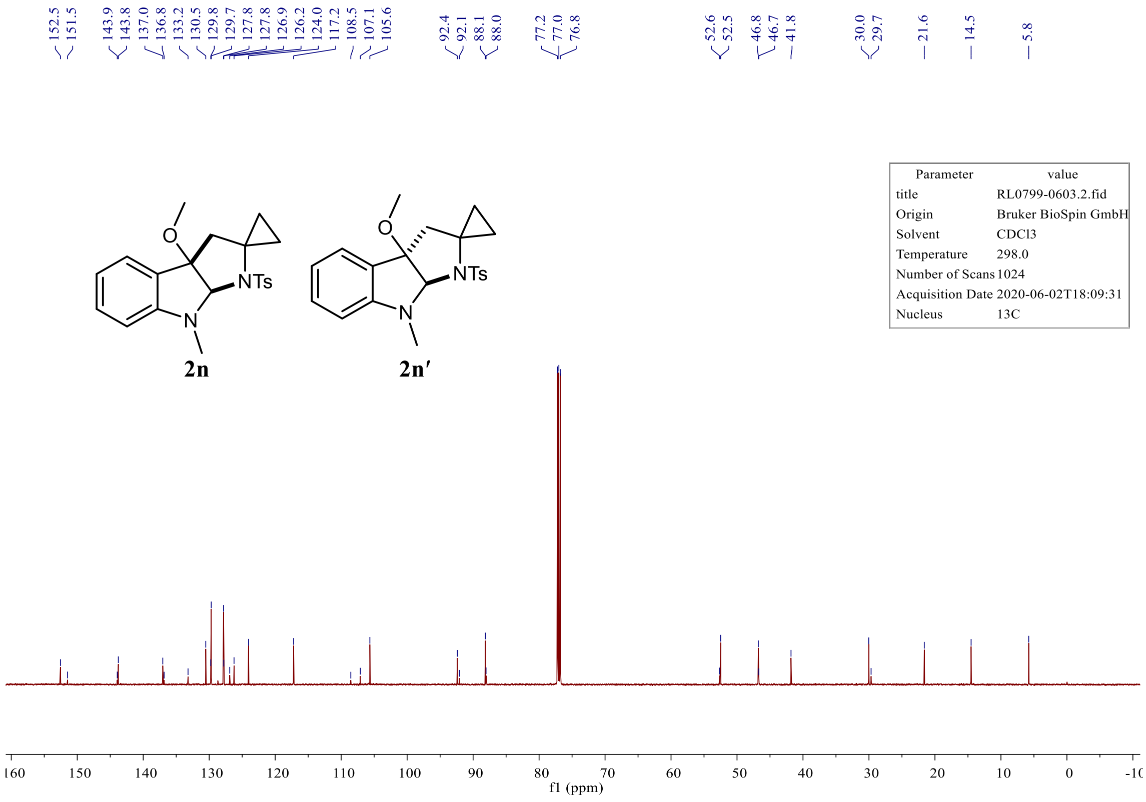
152.5 151.5 143.9 143.8 137.0 136.8 133.2 130.5 129.8 129.7 127.8 127.8 126.9 126.2 124.0 117.2 108.5 107.1 105.6 92.4 92.1 88.1 88.0 77.2 77.0 76.8 52.6 52.5 46.8 46.7 41.8 30.0 29.7 21.6 14.5 5.8
Parameter value
title RL0799-0603.2.fid
Origin Bruker BioSpin GmbH
Solvent CDCl3
Temperature 298.0
Number of Scans 1024
Acquisition Date 2020-06-02T18:09:31
Nucleus 13C
O
NTs
N
2n
2n′
160 150 140 130 120 110 100 90 80 70 60 50 40 30 20 10 0 -10
f1 (ppm)

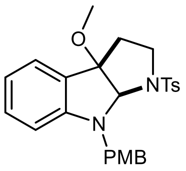

2o

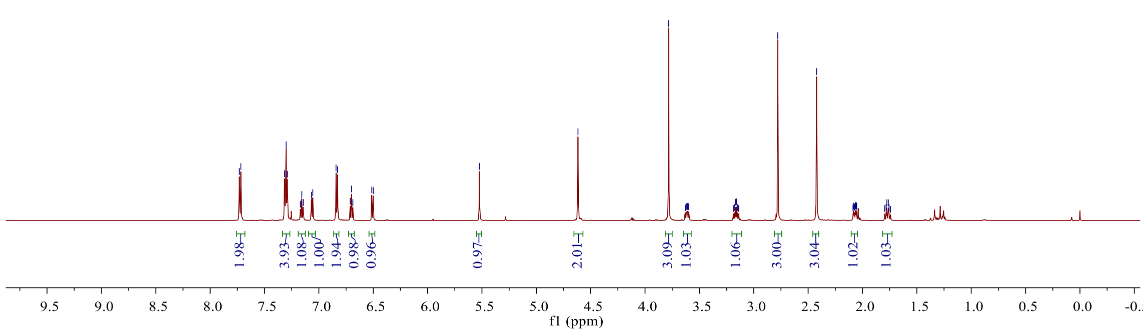

Parameter	value
title	RL-0721.1.fid
Origin	Bruker BioSpin GmbH
Solvent	CDCl3
Temperature	298.0
Number of Scans	16
Acquisition Date	2020-01-13T20:44:04
Nucleus	1H

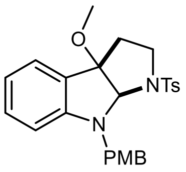

2o

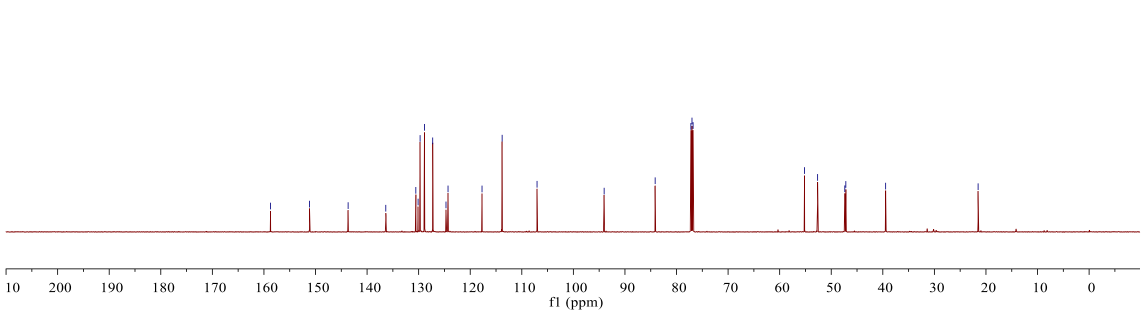

Parameter	value
title	RL-0721.2.fid
Origin	Bruker BioSpin GmbH
Solvent	CDCl3
Temperature	298.0
Number of Scans	1024
Acquisition Date	2020-01-15T06:59:03
Nucleus	13C

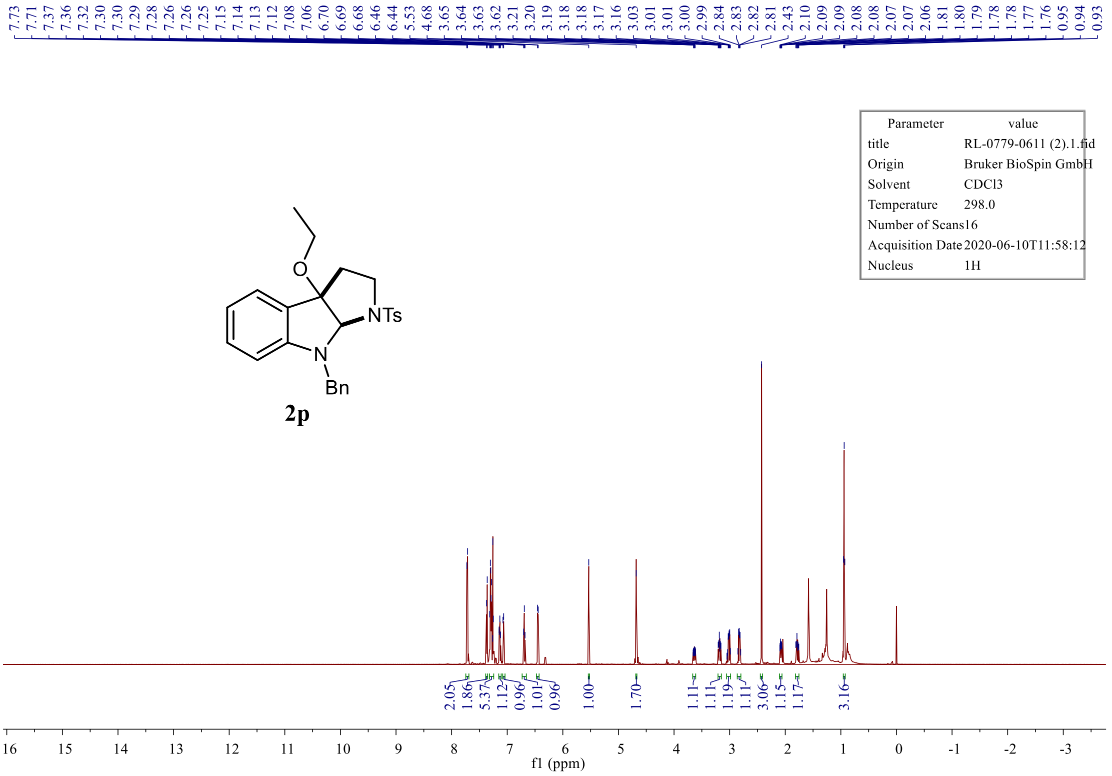
7.73 7.71 7.37 7.36 7.32 7.30 7.30 7.29 7.28 7.26 7.26 7.25 7.15 7.14 7.13 7.12 7.08 7.06 6.70 6.69 6.68 6.46 6.44 5.53 4.68 3.65 3.64 3.63 3.62 3.21 3.20 3.19 3.18 3.18 3.17 3.16 3.03 3.01 3.01 3.00 2.99 2.84 2.83 2.82 2.81 2.43 2.10 2.09 2.09 2.08 2.08 2.07 2.07 2.06 1.81 1.80 1.79 1.78 1.78 1.77 1.76 0.95 0.94 0.93
Parameter value
title RL-0779-0611 (2).1.fid
Origin Bruker BioSpin GmbH
Solvent CDCl3
Temperature 298.0
Number of Scans16
Acquisition Date2020-06-10T11:58:12
Nucleus 1H
O
NTs
N
Bn
2p
2.05 1.86 5.37 1.12 0.96 1.01 0.96 1.00 1.70 1.11 1.11 1.19 1.11 3.06 1.15 1.17 3.16
16 15 14 13 12 11 10 9 8 7 6 5 4 3 2 1 0 -1 -2 -3
f1 (ppm)

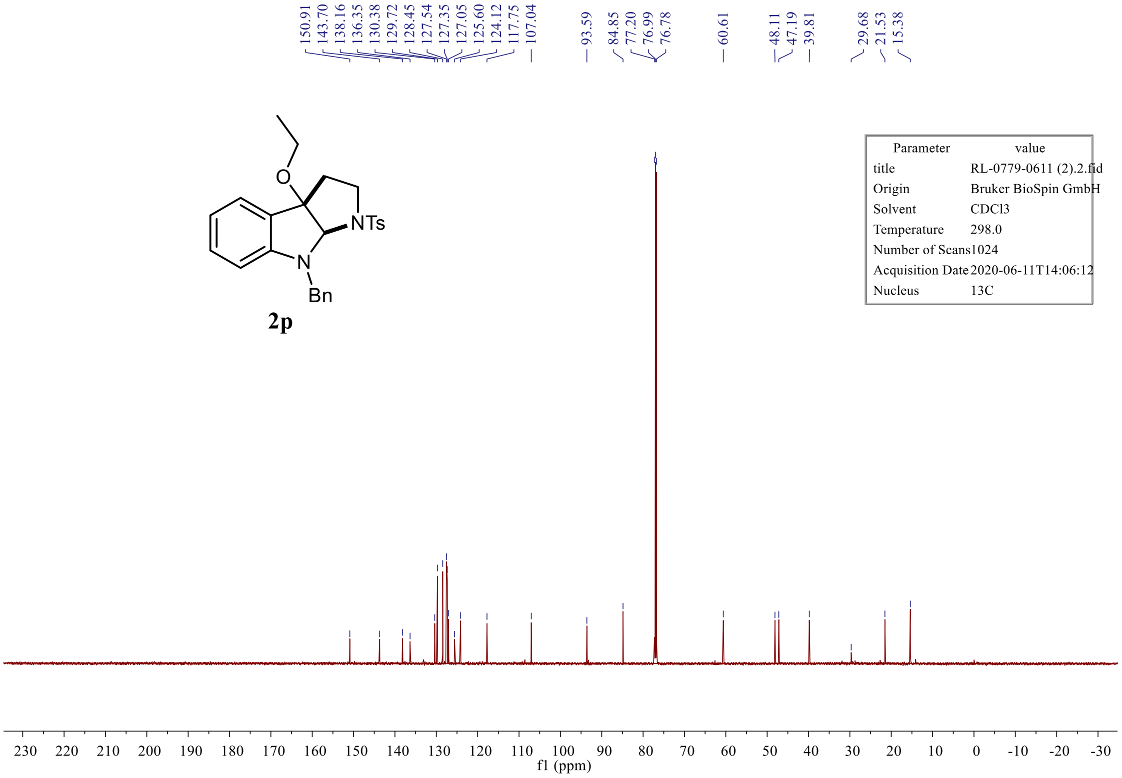
150.91 143.70 138.16 136.35 130.38 129.72 128.45 127.54 127.35 127.05 125.60 124.12 117.75 107.04 93.59 84.85 77.20 76.99 76.78 60.61 48.11 47.19 39.81 29.68 21.53 15.38
Parameter value
title RL-0779-0611 (2).2.fid
Origin Bruker BioSpin GmbH
Solvent CDCl3
Temperature 298.0
Number of Scans1024
Acquisition Date2020-06-11T14:06:12
Nucleus 13C
O
NTs
N
Bn
2p
230 220 210 200 190 180 170 160 150 140 130 120 110 100 90 80 70 60 50 40 30 20 10 0 -10 -20 -30
f1 (ppm)

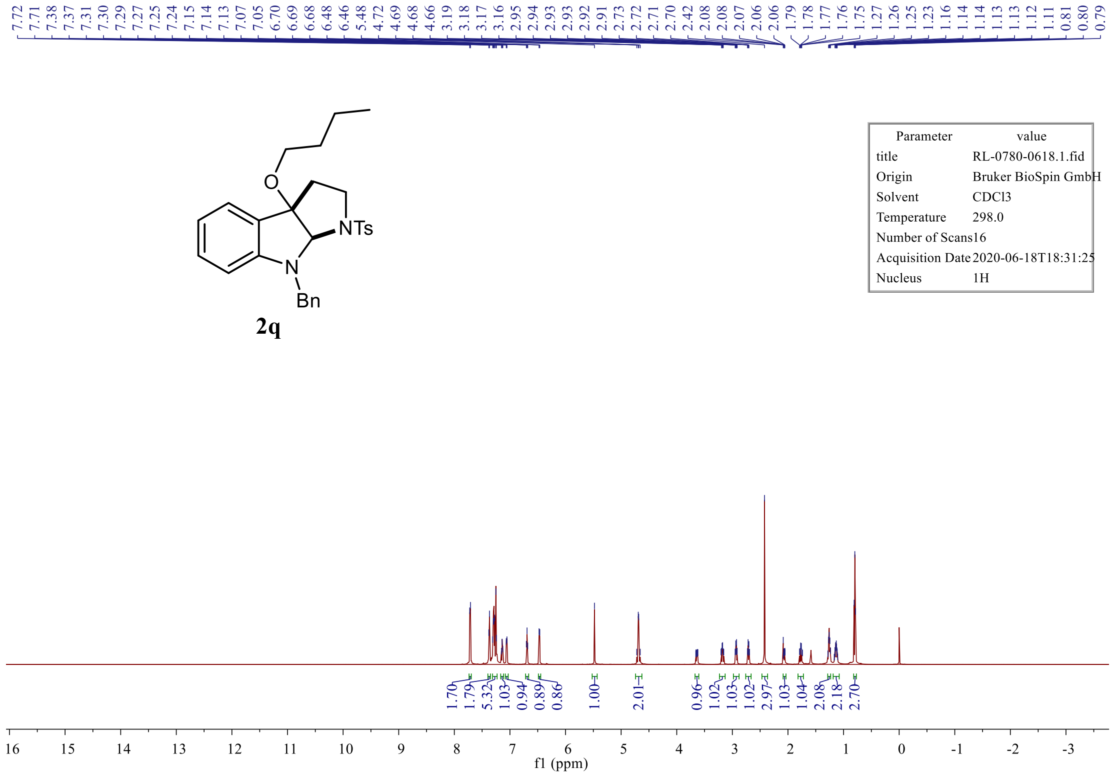
7.72 7.71 7.38 7.37 7.31 7.30 7.29 7.27 7.25 7.24 7.15 7.14 7.13 7.07 7.05 6.70 6.69 6.68 6.48 6.46 5.48 4.72 4.69 4.68 4.66 3.19 3.18 3.17 3.16 2.95 2.94 2.93 2.93 2.92 2.91 2.73 2.72 2.71 2.70 2.42 2.08 2.08 2.07 2.06 2.06 1.79 1.78 1.77 1.76 1.75 1.27 1.26 1.25 1.23 1.16 1.14 1.14 1.13 1.13 1.12 1.11 0.81 0.80 0.79
O
NTs
N
Bn
2q
Parameter value
title RL-0780-0618.1.fid
Origin Bruker BioSpin GmbH
Solvent CDCl3
Temperature 298.0
Number of Scans 16
Acquisition Date 2020-06-18T18:31:25
Nucleus 1H
1.70 1.79 5.32 1.03 0.94 0.89 0.86 1.00 2.01 0.96 1.02 1.03 1.02 2.97 1.03 1.04 2.08 2.18 2.70
16 15 14 13 12 11 10 9 8 7 6 5 4 3 2 1 0 -1 -2 -3
f1 (ppm)

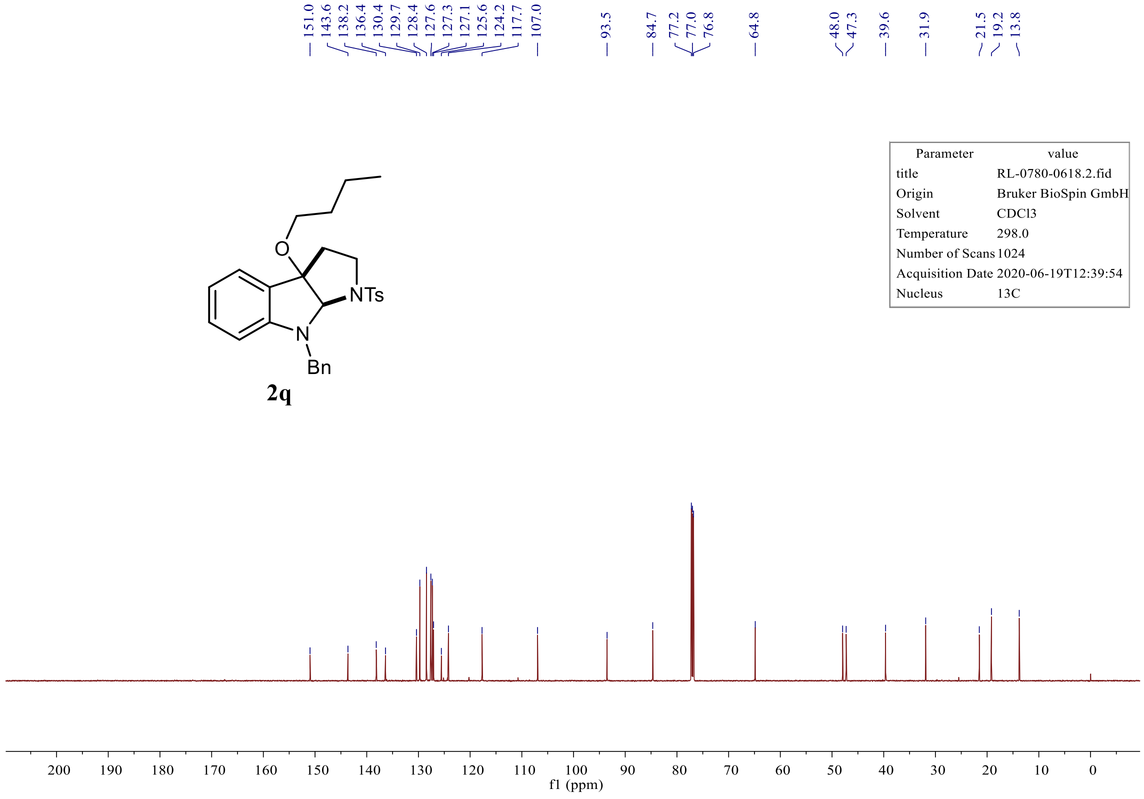
151.0 143.6 138.2 136.4 130.4 129.7 128.4 127.6 127.3 127.1 125.6 124.2 117.7 107.0 93.5 84.7 77.2 77.0 76.8 64.8 48.0 47.3 39.6 31.9 21.5 19.2 13.8
O
NTs
N
Bn
2q
Parameter value
title RL-0780-0618.2.fid
Origin Bruker BioSpin GmbH
Solvent CDCl3
Temperature 298.0
Number of Scans 1024
Acquisition Date 2020-06-19T12:39:54
Nucleus 13C
200 190 180 170 160 150 140 130 120 110 100 90 80 70 60 50 40 30 20 10 0
f1 (ppm)

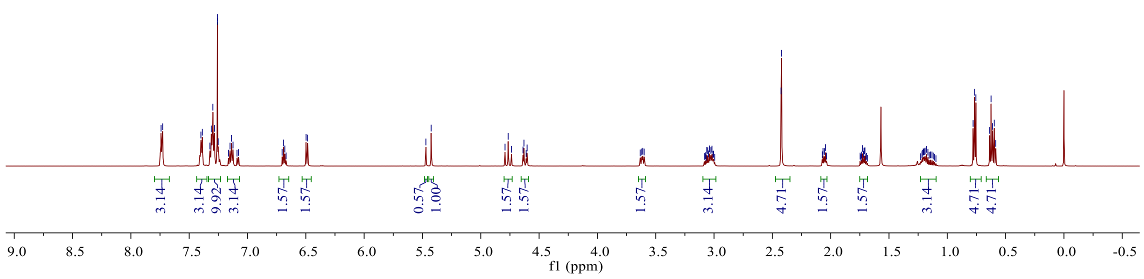

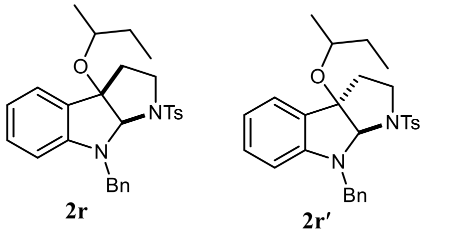

2r **2r′**

Parameter	value
title	RL-0766-0618.1.fid
Origin	Bruker BioSpin GmbH
Solvent	CDCl3
Temperature	298.0
Number of Scans	16
Acquisition Date	2020-06-18T18:35:20
Nucleus	1H

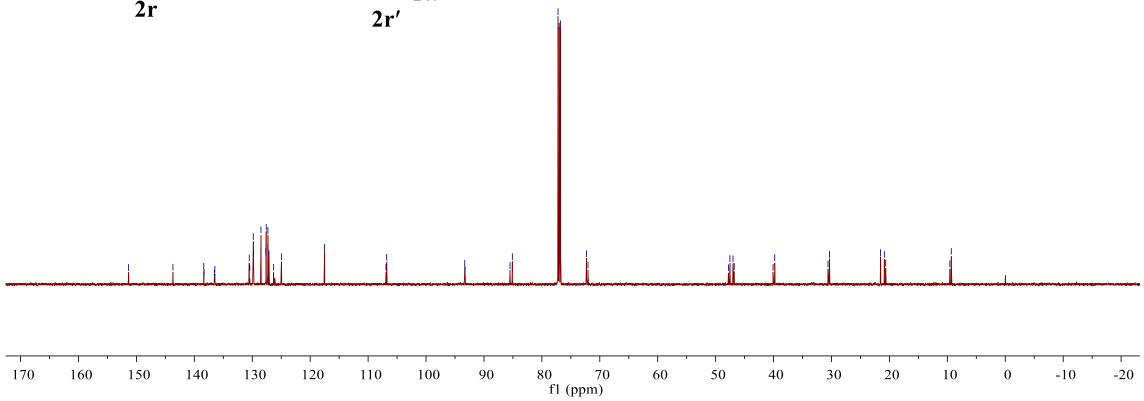

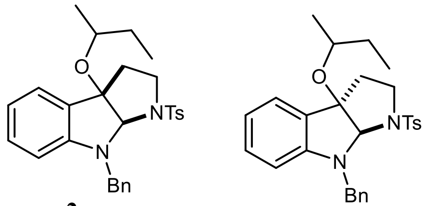

2r **2r′**

Parameter	value
title	RL-0766-0618.2.fid
Origin	Bruker BioSpin GmbH
Solvent	CDCl3
Temperature	298.0
Number of Scans	256
Acquisition Date	2020-06-19T12:56:07
Nucleus	13C

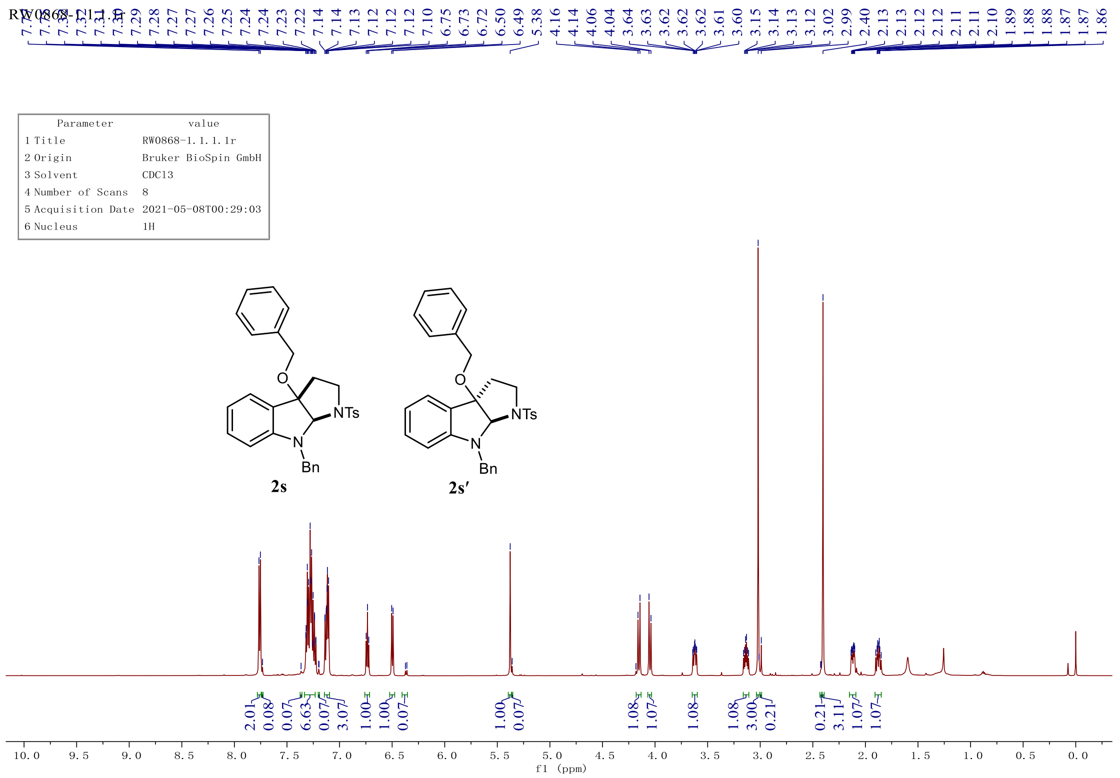
RW0868-1.1.1.1r
Parameter | value
1 Title | RW0868-1.1.1.1r
2 Origin | Bruker BioSpin GmbH
3 Solvent | CDCl3
4 Number of Scans | 8
5 Acquisition Date | 2021-05-08T00:29:03
6 Nucleus | 1H
7.31 7.31 7.29 7.28 7.27 7.27 7.26 7.25 7.24 7.24 7.23 7.22 7.14 7.14 7.13 7.12 7.12 7.12 7.10 6.75 6.73 6.72 6.50 6.49 5.38 4.16 4.14 4.06 4.04 3.64 3.63 3.62 3.62 3.62 3.61 3.60 3.15 3.14 3.13 3.12 3.02 2.99 2.40 2.13 2.13 2.12 2.12 2.11 2.11 2.10 1.89 1.88 1.87 1.87 1.86
NTs
O
N
Bn
2s
2s′
2.01 0.08 0.07 6.63 0.07 3.07 1.00 1.00 0.07 1.00 0.07 1.08 1.07 1.08 1.08 3.00 0.21 0.21 3.11 1.07 1.07
10.0 9.5 9.0 8.5 8.0 7.5 7.0 6.5 6.0 5.5 5.0 4.5 4.0 3.5 3.0 2.5 2.0 1.5 1.0 0.5 0.0
f1 (ppm)

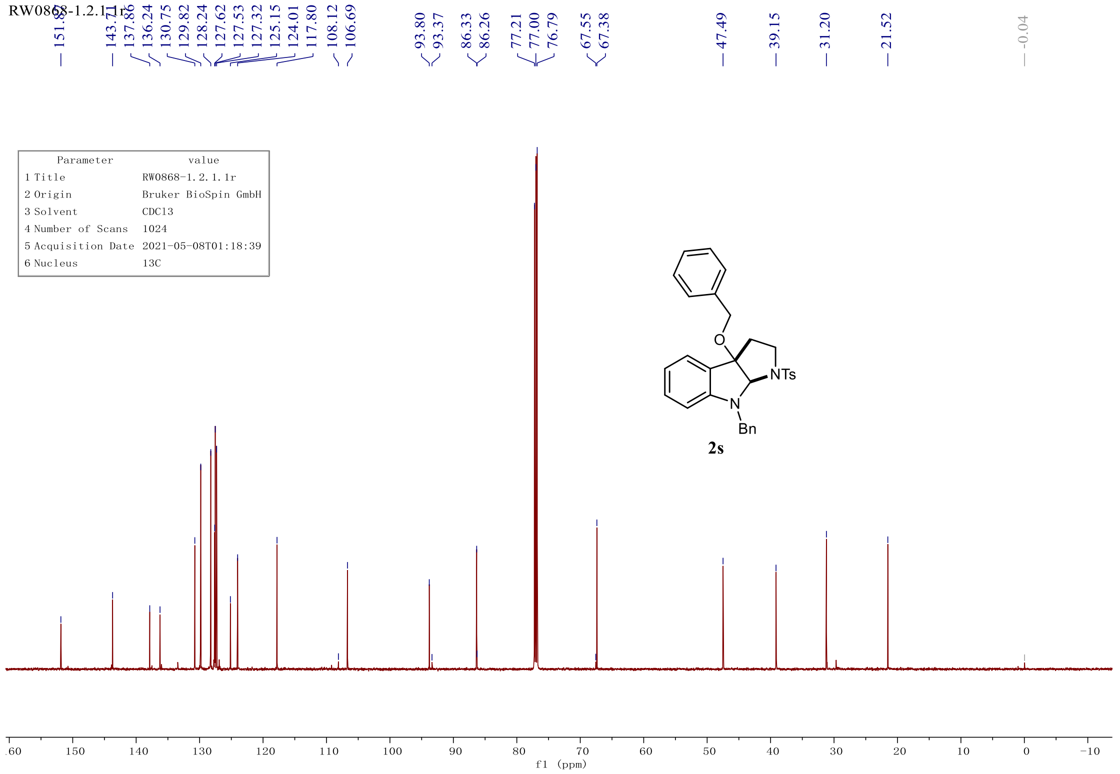
RW0868-1.2.1.1r
Parameter | value
1 Title | RW0868-1.2.1.1r
2 Origin | Bruker BioSpin GmbH
3 Solvent | CDCl3
4 Number of Scans | 1024
5 Acquisition Date | 2021-05-08T01:18:39
6 Nucleus | 13C
143.71 137.86 136.24 130.75 129.82 128.24 127.62 127.53 127.32 125.15 124.01 117.80 108.12 106.69 93.80 93.37 86.33 86.26 77.21 77.00 76.79 67.55 67.38 47.49 39.15 31.20 21.52 -0.04
NTs
O
N
Bn
2s
160 150 140 130 120 110 100 90 80 70 60 50 40 30 20 10 0 -10
f1 (ppm)

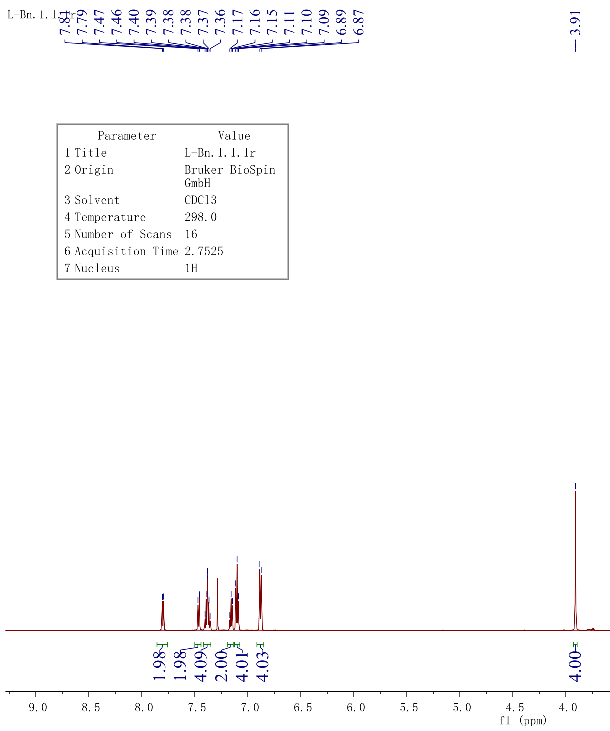

Parameter	Value
1 Title	L-Bn.1.1.1r
2 Origin	Bruker BioSpin GmbH
3 Solvent	CDCl3
4 Temperature	298.0
5 Number of Scans	16
6 Acquisition Time	2.7525
7 Nucleus	1H

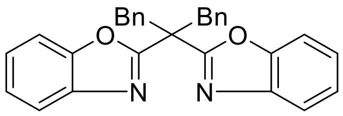

L4

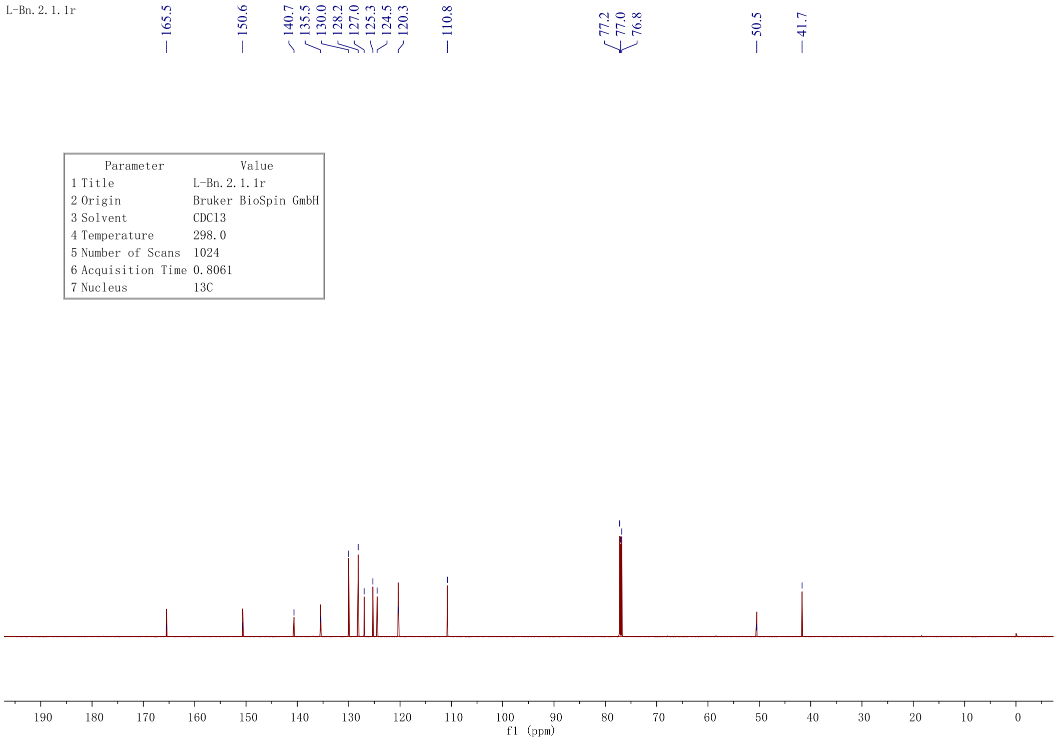

Parameter	Value
1 Title	L-Bn.2.1.1r
2 Origin	Bruker BioSpin GmbH
3 Solvent	CDCl3
4 Temperature	298.0
5 Number of Scans	1024
6 Acquisition Time	0.8061
7 Nucleus	13C

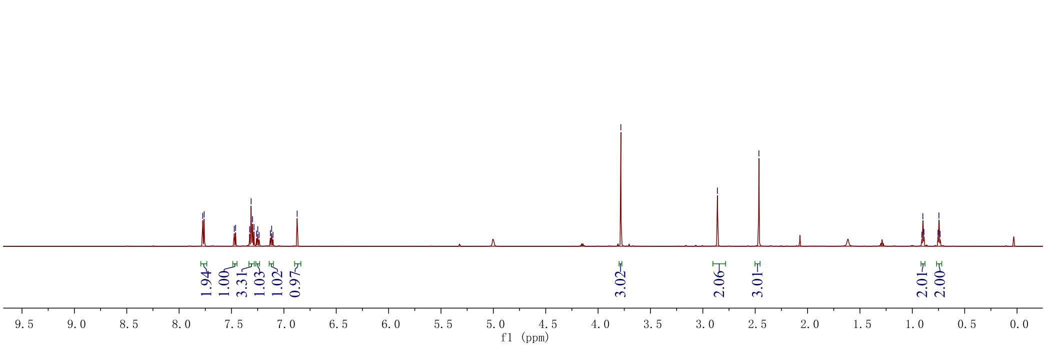

Parameter	Value
1 Title	Rs-0482.1.1.1r
2 Origin	Bruker BioSpin GmbH
3 Solvent	CDCl3
4 Temperature	298.0
5 Number of Scans	16
6 Acquisition Date	2020-01-08T04:07:55
7 Nucleus	1H

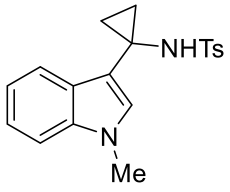

1n

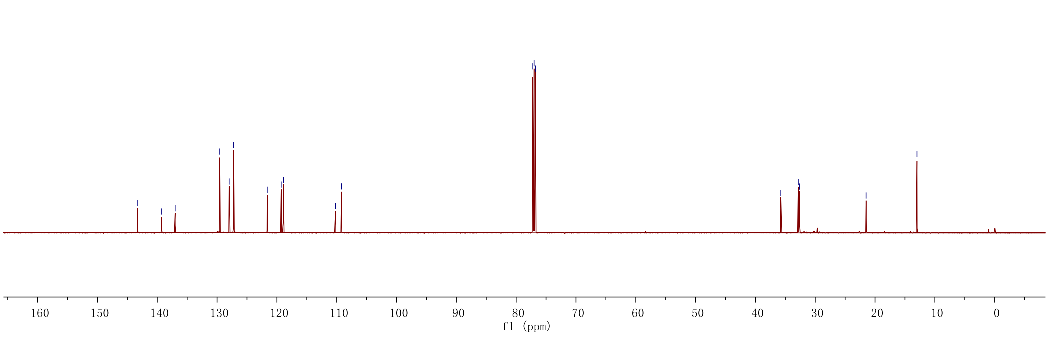

Parameter	Value
1 Title	RS-0482.2.1.1r
2 Origin	Bruker BioSpin GmbH
3 Solvent	CDCl3
4 Temperature	298.0
5 Number of Scans	1024
6 Acquisition Date	2020-07-23T07:12:11
7 Nucleus	13C

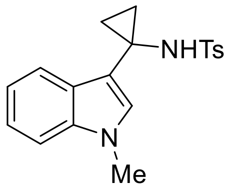

1n

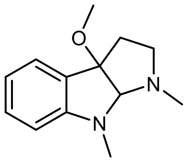

CPC-1

Parameter	value
title	RL0813C.1.fid
Origin	Bruker BioSpin GmbH
Solvent	CDCl3
Temperature	298.0
Number of Scans	16
Acquisition Date	2020-06-01T21:36:54
Nucleus	1H

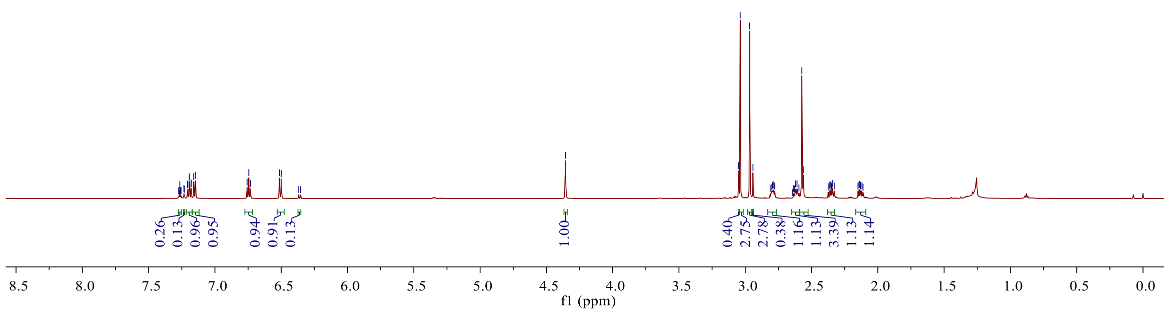

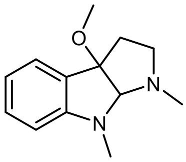

CPC-1

Parameter	value
title	RL0813.1.fid
Origin	Bruker BioSpin GmbH
Solvent	CDCl3
Temperature	298.1
Number of Scans	32
Acquisition Date	2020-06-03T18:39:20
Nucleus	13C

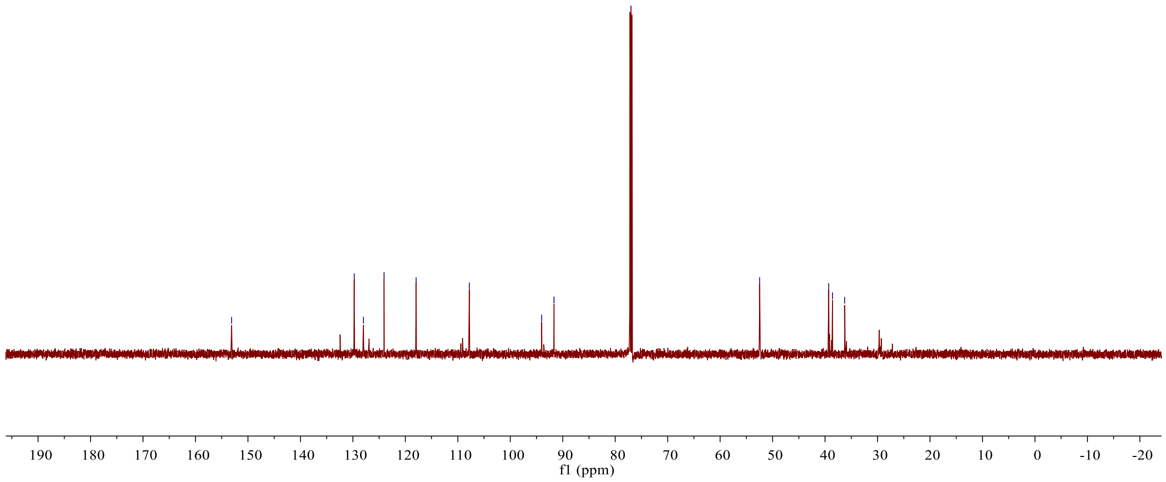

Supplement: RA-011-D1RA02679H-s001 [file RA-011-D1RA02679H-s001.pdf]
